# Organocyclophosphazenes and Materials Based on Them for Pharmaceuticals and Biomedicine

**DOI:** 10.3390/biom15020262

**Published:** 2025-02-11

**Authors:** Pavel Yudaev, Anton Tupikov, Evgeniy Chistyakov

**Affiliations:** Chemical Technology of Russia, Mendeleev University, Miusskaya Sq. 9, 125047 Moscow, Russiaewgenijj@rambler.ru (E.C.)

**Keywords:** phosphazenes, physiologically active substance, anticancer drug, cytotoxicity, cancer, oncology, antimicrobial action, biosensors, gels, dental materials

## Abstract

This review examines representatives of organocyclophosphazenes that can act against tumor cells of the ovaries, prostate gland, mammary gland, and colon, etc., and have antimicrobial action against mycobacteria *M. tuberculosis*, Gram-positive bacteria *B. cereus*, Gram-negative bacteria *K. pneumaniae*, fungi of the genus *Candida*, and other microorganisms. Cyclomatric phosphazenes can be used as carriers of physiologically active substances and in the field of detection, as well as gels for wound surgery and drug delivery platforms. In gels, cyclophosphazenes are used as cross-linking agents. Cyclophosphazenes containing multiple bonds in organic radicals are proposed to be used in dentistry as additives to basic dental compositions. Particular attention in the review is paid to the cytotoxic and antimicrobial action of materials containing cyclophosphazenes and their advantages over commercial physiologically active substances. The review presents the prospects for the practical application of cyclophosphazenes containing various functional groups (chalcone, anthraquinone, pyrrolidine, morpholine, and ferrocene, etc.) in pharmaceuticals. The review may be of interest to researchers working in the field of organoelement chemistry, medicine, and pharmacy.

## 1. Introduction

There are a number of problems in the field of pharmacology and medicine. Physiologically active substances (PASs) used in oncological diseases have a number of disadvantages. These include a low therapeutic range, poor solubility in water due to the hydrophobicity of some cytostatics (e.g., paclitaxel), cytotoxicity in relation to normal human cells, and a wide range of side effects. For example, doxorubicin causes cardiotoxicity [1], leading to cardiomyopathy and heart failure [2]. Paclitaxel leads to neuropathy [3] and complex platinum compounds (cisplatin, nedaplatin, and carboplatin) to nephrotoxicity, nausea, and vomiting [4]. Also, resistance to anticancer drugs can be developed, for example, to anthraquinone derivatives such as mitoxantrone [5] and commercial antibiotics used in relation to microbial infections [6], a technique that requires the correct choice of treatment strategy and PAS concentration during treatment.

In the field of regenerative medicine, the problem of the cytotoxicity of wound polymeric materials is known, caused by the toxicity of gelling agents such as glutaraldehyde [7], glyoxal, and diethyl squarate, etc.

The use of nanoparticles in medical devices is also complicated by the uneven distribution of silver nanoparticles in the polymer matrix and their aggregation, leading to a deterioration in their antimicrobial action [8], a deterioration in physical and mechanical properties, especially of dental restorative materials [9,10], and the leaching of nanoparticles from the compositions leads to the intoxication of the surrounding tissues.

Another challenge is the creation of chemical sensors that are selective and sensitive to the analyte in biochemical samples such as sweat and saliva, etc., [11], acting as electrochemical sensors of neurotransmitters for the treatment of neurodegenerative diseases.

To solve the above problems, the use of organocyclophosphazenes (OCPs), which are a class of organic–inorganic hybrid materials with chemical lability, is promising. Unlike silver nanoparticles, which are highly cytotoxic and cause side effects in patients (argyria, argyrosis, etc.), OCPs are potentially non-toxic to humans.

Varying the organic substituents at the phosphorus atoms in phosphazene cycles allows for obtaining compounds with a given structure and a set of valuable properties.

OCPs have proven themselves in many areas of science and technology, in particular, in the field of analytics for the selective determination of heavy metal ions in biochemical samples [12]; as catalysts for polymerization [13,14] and the conversion of carbon dioxide into methanol [15]; in the field of extraction for the recovery of heavy [16], alkali [17], and rare earth metals [18]; and for the creation of lighting and visualization devices [19]. Metal complexes and coordination polymers based on OCPs are of considerable interest in the field of creating fire-retardant materials [20] and wastewater purification from organic dyes [21].

OCPs increase the heat resistance and fire resistance of polymer binders, such as epoxy [22,23], benzoxazine [24], ethylene copolymers [25], and textiles [26,27], and also improve the chemical resistance of polyester and polyurethane coatings [28].

OCPs, unlike linear polyphosphazenes, have a monodisperse main chain, which facilitates the control of their molecular weight and purity, while for linear polyphosphazenes, control of the molecular weight is only possible for short polymer chains (up to 50 units) [29]. The ease of molecular weight control and the availability of the original chlorocyclophosphazenes are also advantages of cyclophosphazenes, especially when used in pharmaceuticals. For example, based on OCPs with three or four phosphazene units, dendrimeric structures have been obtained that can act as PASs with antimicrobial and antiproliferative action [30], as well as PAS nanocarriers [31].

In addition, nitrogen atoms in phosphazene rings are capable of coordinating metals with cytostatic action, such as gold or silver [32].

To date, review articles have been published on linear phosphazenes in the form of nanoparticles and gels [33,34,35] and cyclophosphazenes acting as anticorrosive materials [36]. However, OCPs with antiproliferative and antimicrobial action for use in pharmaceuticals and biomedicine have not yet been comprehensively studied in the scientific literature.

This review presents the advantages, disadvantages, and prospects for future use of OCPs containing various functional groups at the phosphorus atoms of the phosphazene ring in various fields of medicine. A comparative analysis of their antitumor and antimicrobial properties is carried out and recommendations are given for the further research of OCPs necessary for their introduction into clinical practice.

## 2. OCPs with Antitumor Effect

Cancer is difficult to detect and difficult to treat. Today, the main methods of treating oncological diseases are chemotherapy, radiation therapy, and surgical methods. Chemotherapeutic drugs are administered in high doses, and they cause many side effects, harming not only tumor tissues, but also healthy ones [37].

Cyclophosphazenes, unlike commercial cytostatics, are non-toxic to the human body. Among cyclophosphazenes with antitumor activity, we can distinguish cyclophosphazenes containing chalcone groups, nitrogen heterocycles, peptide bonds, anthraquinones, parabens, and ferrocene fragments.

### 2.1. OCPs Containing Chalcone Groups

Cyclophosphazenes containing chalcone groups have antitumor activity against human ovarian and prostate cancer cells. The mechanism of their antitumor activity was examined in the works of researchers from Turkey [38,39].

**Figure 1:**
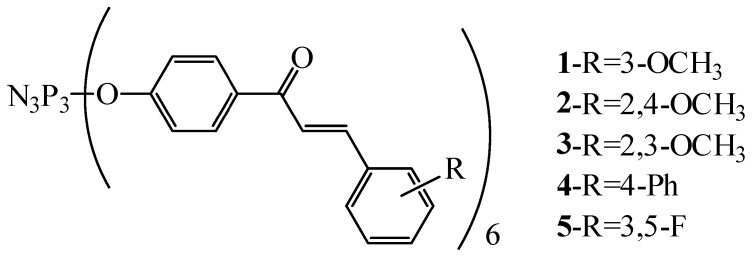
OCPs with chalcone groups.

In particular, in the work [38], five hexasubstituted cyclophosphazenes with chalcone groups were synthesized (Figure 1) and it was established that they have activity against human ovarian cancer cells (cell line A2780) and prostate cancer cells (cell lines PC-3 and LNCaP), since cyclophosphazenes reduced their viability by 70–90% after 24 h of observation.

According to the authors, the antitumor activity of phosphazenes is due to the binding to the β-subunit of the tubulin protein, from which the microtubules of cells involved in mitosis are built, using hydrogen bonds, π–π stacking, and π–cation interactions, which inhibit the process of cancer cell division. The authors established which amino acids the chalcone fragments bind to (Table 1).

The authors note that hexasubstituted phosphazenes reduce the viability of cancer cells to a greater extent than di- and tetrasubstituted phosphazenes containing the same chalcone groups at the phosphazene ring (Figure 1). Unfortunately, the interaction of cyclophosphazenes containing chalcone groups with DNA fragments of cancer cells was not studied in [38].

**Figure 2:**
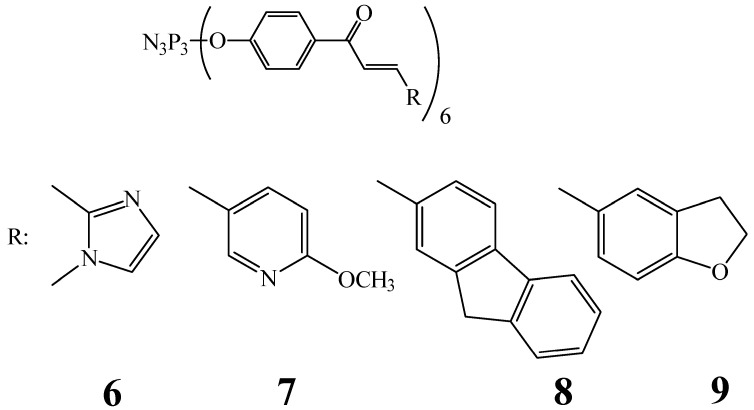
OCPs containing chalcone fragments and cycles.

The same scientific group in the work [39], using the molecular docking method, investigated the process of inhibition of enzymes associated with the DNA of cancer cells (cell lines A2780, PC-3, and LNCaP) and direct interaction with the DNA of cancer cells for cyclophosphazenes containing chalcone fragments and heterocycles (compounds **6**–**9**, Figure 2).

The authors found that, compared to low-molecular-weight chalcones, cyclophosphazenes have higher binding rates to the proteins Bcl-2, p53, Caspase-3, and Src kinase, due to their larger molecular size. The authors modeled interactions with the proteins’ amino acids and showed that the chalcone aromatic ring binds, in particular, to arginine and PRD (histidine–proline) repeat via non-covalent π–cationic/π–anionic interactions.

In addition, in work [39], it was established that due to the flexibility of phosphazene structures **6**–**9**, they are easily integrated into the DNA chain and interact with the components of the minor groove of DNA (thymine, guanine, cytosine, and sugar–phosphate backbones) due to non-covalent interactions, in particular π–cation, π–anion, π–lone pair, π–π stacking, and π–alkyl interactions. Due to the wide range of these interactions, phosphazenes cause damage to the DNA of cancer cells.

The authors of the work [39] also suggest that phosphazenes **6**–**9** do not penetrate the blood–brain barrier (BBB) due to their high polarity. This fact is an advantage of cyclophosphazenes over low-molecular chalcones, since the BBB prevents them from passing from the blood to the brain, and the permeability of the BBB for drugs is important mainly in the treatment of diseases of the central nervous system. However, the authors did not conduct in vivo studies of the introduction of the substance into the bloodstream or its determination in the brain tissue of a laboratory animal to determine the permeability of the BBB by phosphazenes **6**–**9**.

Thus, cyclophosphazenes containing chalcone fragments are promising PASs for cancer treatment due to their ability to bind to cellular DNA and their cytotoxicity towards cancer cells in low concentrations (no more than 100 μmol/L). However, additional studies of their cyto- and genotoxicity towards normal cells (e.g., mouse fibroblasts L929) and the selection of optimal concentrations are required. In addition, the compounds obtained in [39] are not soluble in water, and their synthesis and purification are multi-stage and relatively complex, which may limit their use in pharmaceuticals.

### 2.2. OCPs Containing N-Heterocycles

**Figure 3:**
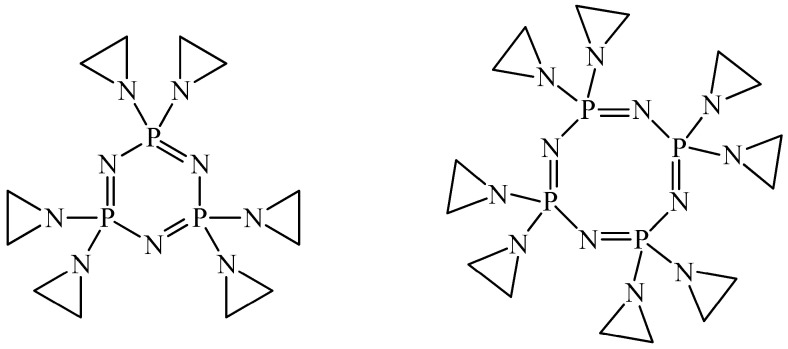
OCPs with aziridine fragments.

Since the late 1970s, it has been known that cyclophosphazenes containing aziridine fragments (Figure 3) have high solubility in water, and antitumor activity against leukemia and melanoma [40]. This makes them promising for use as cytostatics. However, a disadvantage of aziridine derivatives of cyclophosphazenes is their ability to accumulate in bone marrow and react with cellular nucleophiles [41].

**Figure 4:**
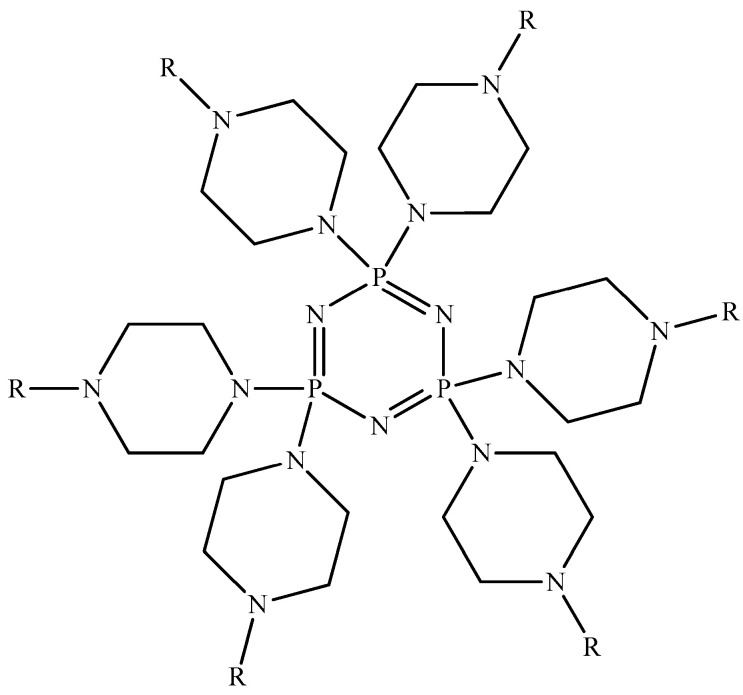
OCPs containing piperazine N-heterocycles (R = CH_3_ or C_2_H_5_ or C_6_H_5_).

Hexasubstituted cyclophosphazenes containing N-methyl-, N-ethyl-, and N-phenylpiperazine fragments (Figure 4) can also be used as cytostatics. The modeling of their interactions using molecular docking with proteins BRAF kinase, thymidylate synthase TS, and ribonucleotide reductase RNR and DNA showed that they bind better to proteins and the minor groove of DNA compared to the commercial cytostatic doxorubicin [42].

In addition, the authors of [42] found that the derivative with the C_6_H_5_ group in the piperazine ring interacts better with minor centers in DNA compared to derivatives with CH_3_ and C_2_H_5_ groups. Unfortunately, there is no explanation for this fact in the work. In addition, it would be useful to conduct studies on the effect of cyclophosphazenes on the viability of normal and cancer cells.

In contrast to cyclophosphazenes with piperazine rings, the cytotoxic effect of cyclophosphazenes containing pyrrolidine, piperidine, morpholine, and 1,4-dioxa-8-azaspiro [4,5]decane (DASD) rings has been studied in relation to both cancer and normal cells [43,44,45].

**Figure 5:**
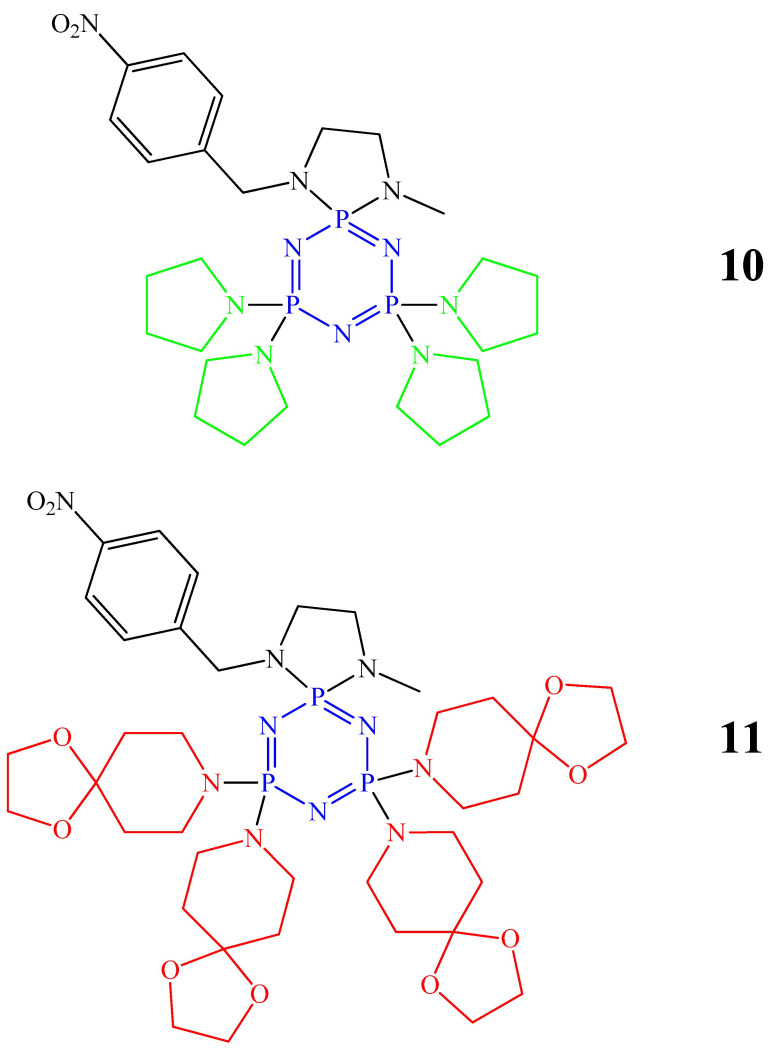
OCPs containing pyrrolidine rings (marked in green) and DASD (marked in red).

The disadvantage of cyclophosphazenes containing pyrrolidine, piperidine, morpholine, and DASD cycles is their low solubility in water. To improve their solubility in water, additional fragments are attached to the nitrogen atom of the organic spirocycle (Figure 5). For example, in work [43], a 4-nitrobenzyl group was used as a side fragment at the nitrogen atom of the spirocycle, and in works [44,45], a 2-furanylmethyl group was used. In the study [43], it was found that cyclophosphazene containing pyrrolidine rings and cyclophosphazene containing DASD rings (compounds **10**, **11**, Figure 5) have a cytotoxic effect on MDA-MB-231 cancer cells at low concentrations (no more than 50 μmol/L), and that OCP **10** was less cytotoxic than OCP **11**. Unfortunately, the authors do not provide an explanation for this fact.

However, both OCP **10** and **11** were also cytotoxic to normal cells (L929 fibroblasts).

However, the cytotoxic effect and apoptotic effect of the above cyclophosphazenes on fibroblasts was less than that of the commercial antitumor drug cisplatin.

**Figure 6:**
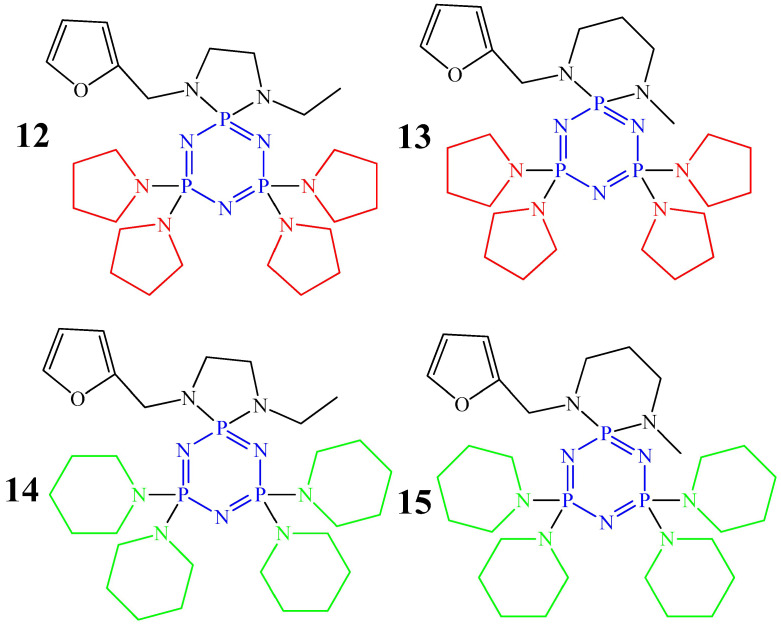
OCPs containing pyrrolidine (compounds **12**, **13**) and piperidine (compounds **14**, **15**) rings.

**Figure 7:**
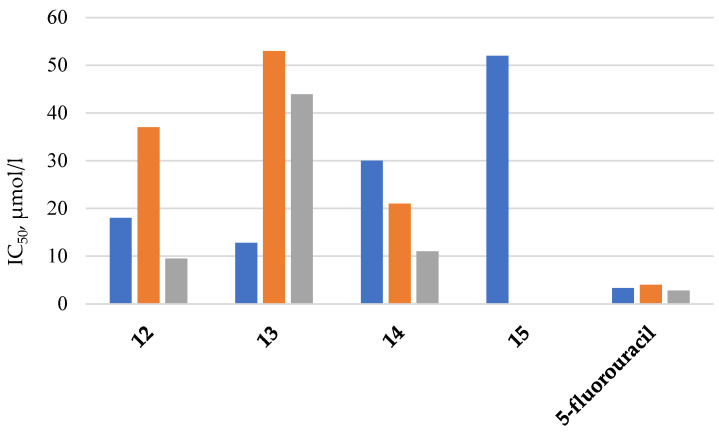
IC_50_ values after 48 h (MTT experiment) for three cell lines (blue column—MCF-7, orange column—HT-29, and gray column—PC-3). For compound **15**, the IC_50_ value could not be calculated because the cell viability was higher than in the control group (only medium).

Work [44] presents cyclophosphazenes containing one spirocycle with a furanyl fragment and pyrrolidine or piperidine substituents as the remaining substituents at the phosphorus atoms (compounds **12**–**15**, Figure 6). These compounds are capable of inhibiting the growth of human breast cancer cells (MCF-7 cell line), prostate cancer PC-3, and colon cancer (HT-29 cell line). According to the values of the 50% inhibitory concentration IC_50_, which is an indicator of the chemosensitivity of cancer cells (Figure 7), compounds **12** and **13** were the most effective against MCF-7 cancer cells, and compounds **12** and **14** were the most effective against HT-29 and PC-3 cells. However, the IC_50_ values for all compounds were higher than for 5-fluorouracil (Figure 7), which is used in chemotherapy for colon and breast cancer, which is a disadvantage of cyclophosphazenes, since more cytostatics are required with a higher IC_50_ value.

Further studies [44] showed that compounds **12**–**15** have anti-migration activity, i.e., they suppress the migration and invasion of cancer cells, and the strongest effect was observed in relation to colon cancer cells. Unfortunately, studies [43,44] do not contain studies of the interaction of the synthesized compounds with various sections of cancer cell DNA, which is important for understanding the mechanism of antitumor action.

### 2.3. OCPs Containing Paraben and Anthraquinone Moieties

**Figure 8:**
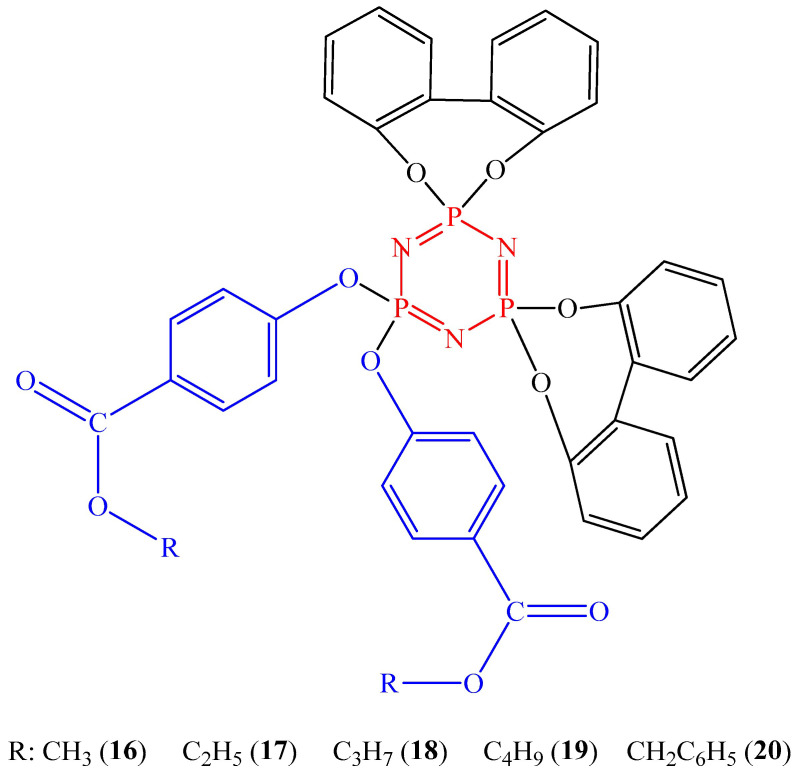
OCPs containing paraben fragments.

Cyclotriphosphazenes containing two paraben moieties (compounds **16**–**20**, Figure 8) and two biphenyl spirocycles per molecule showed cytotoxic activity in vitro against MCF-7 breast cancer cells and colorectal adenocarcinoma cancer cells (DLD-1 cell line) [46]. The viability of cancer cells was less than 50%. The lowest concentration value of 12.5 μmol/L was obtained for compound **17** and MCF-7 cancer cells. However, for compounds **16**, **18**, **19**, and **20**, the concentration values for both cell types were at least 50 μmol/L.

Unfortunately, the work lacks explanations of the indicated differences in minimum concentrations, as well as of the mechanism of the cytotoxic action of the synthesized phosphazenes, studies of their effect on non-cancerous cells, information on the proliferation of cancer cells, the cell cycle, and apoptosis.

**Figure 9:**
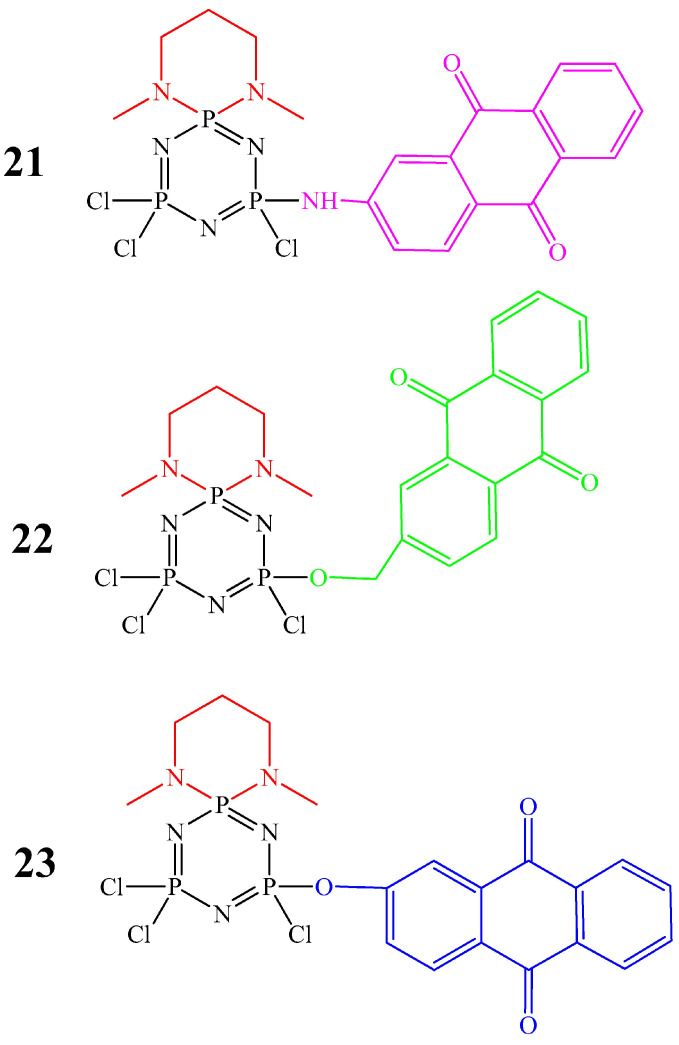
OCPs containing anthraquinone fragments.

In contrast to paraben-containing cyclophosphazenes, derivatives with anthraquinone moieties have been studied for their selective activity against cancer cells. In particular, cyclophosphazenes **21**–**23** (Figure 9) were much more active against non-small cell lung carcinoma cells (A549 and H1299 cell lines) compared to non-neoplastic mesothelial cells (MeT-5A cell line) [47]. In addition, compound **22** showed antitumor activity at a lower concentration (50 μmol/L) compared to compounds **21** and **23**, 2-aminoanthraquinone, 2-hydroxymethylanthraquinone, and 2-hydroxyanthraquinone (100 μmol/L concentration).

Unfortunately, in work [47], there is no explanation as to why compound **22** has a better antitumor effect, in addition, the mechanism of the antitumor effect and nuclear delivery are missing. Nuclear delivery is specifically needed for cancer therapy involving drugs interacting with DNA (no need if an antitumor OCP turns out to be, e.g., a microtubule inhibitor). In our opinion, a possible mechanism is translocation into the cell nucleus through nuclear pores, since the size of OCP molecules, even with very large substituents, does not exceed 10 nm, intercalation into DNA in vitro, or the formation of reactive oxygen species ROS and hydrogen peroxide, as in the commercial drug doxorubicin containing an anthraquinone fragment [48].

In general, the disadvantage of cyclophosphazenes containing paraben or anthraquinone fragments is the difficulty of isolating individual substances using column chromatography and their insolubility in water.

### 2.4. OCPs with Peptide Bonds

**Figure 10:**
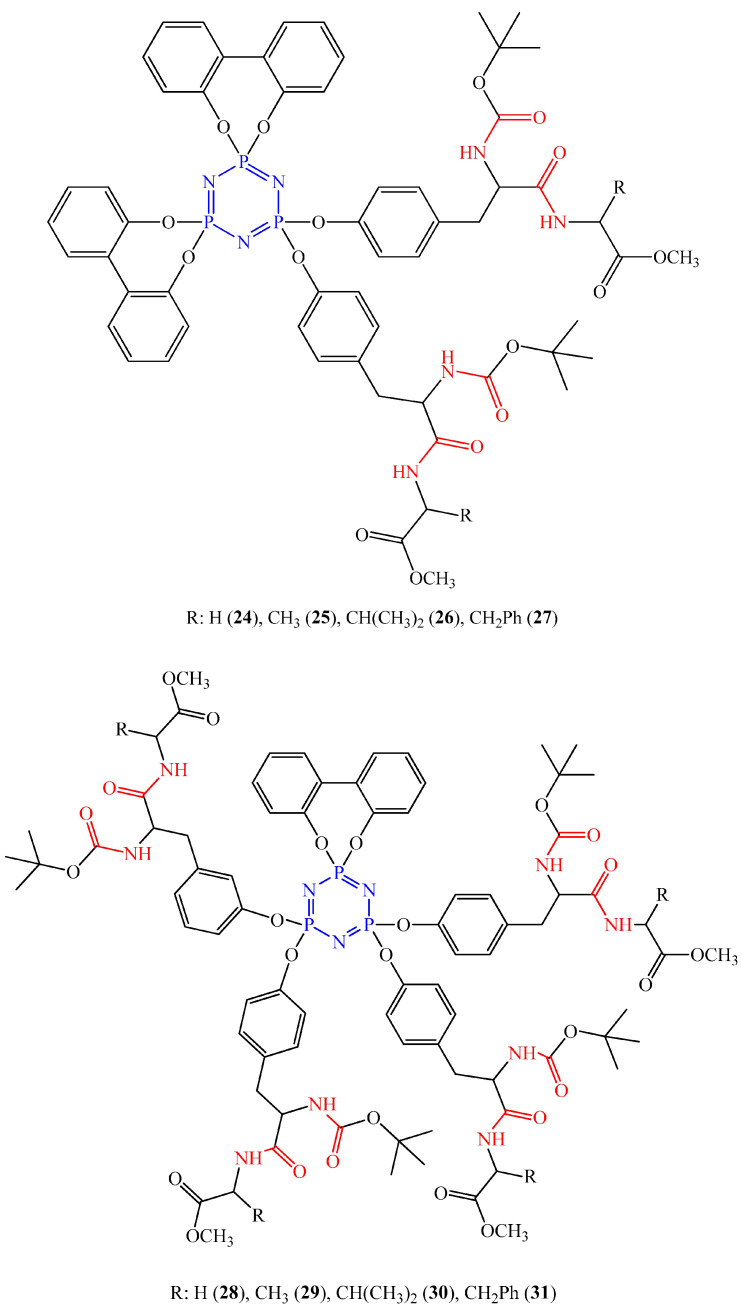
Di- and tetradipeptide-substituted OCPs. The peptide bond is shown in red.

Di- and tetradipeptide-substituted spirocyclotriphosphazenes (compounds **24**–**31**, Figure 10) exhibit cytotoxic activity against human breast cancer cells MCF-7, ovarian cancer cells (cell line A2780), prostate cancer cells (cell line PC-3), and colon cancer cells (cell line Caco-2). The authors of [49] found that the presented OCPs cause a dose-dependent decrease in the viability of the indicated cancer cells, with dipeptide-containing phosphazenes **24**–**27** being more effective against cancer cells than tetrapeptide-containing OCPs **28**–**31**, as evidenced by lower IC_50_ values. The effect on normal breast epithelial cells (cell line MCF-10A) was statistically insignificant.

The authors explained that the decrease in the viability of cancer cells occurs via the mechanism of damage to cellular DNA, which was confirmed by comet analysis. According to the authors, not only peptide bonds, but also the planar biphenyl structure, aromatic rings of Boc-L-Tyrosine and phenylalanine, participate in the interaction with DNA nucleotides.

**Figure 11:**
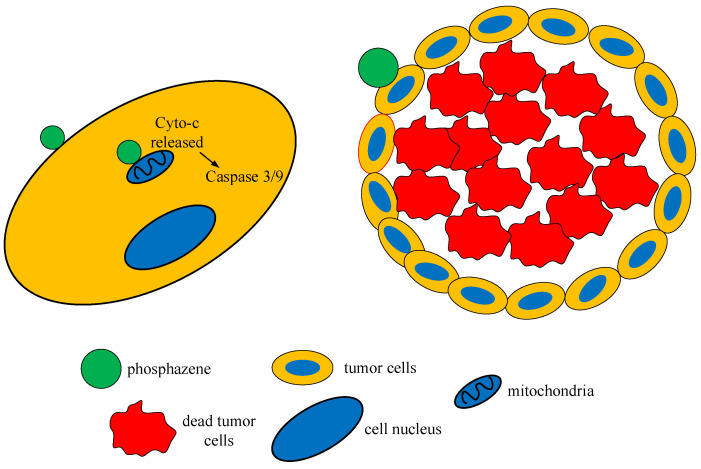
Mechanism of cytotoxic action of OCPs containing peptide bonds.

Dipeptide-containing and terapeptide-containing phosphazenes, like antitumor peptides, can interact with the negatively charged cell membrane of cancer cells through electrostatic interactions, and lead to the destruction of the cell membrane of cancer cells and the leakage of cytoplasmic contents [50,51]. Another possible mechanism of action of the synthesized compounds is the induction of apoptosis. Phosphazenes containing a peptide bond destroy the mitochondrial membrane of tumor cells, release cytochrome C (Cyto-C), and activate the caspase apoptotic pathway (Figure 11).

The advantage of cyclophosphazenes containing peptide bonds, in our opinion, is their high specificity and fewer possible side effects compared to traditional chemotherapeutic drugs. In addition, unlike cyclophosphazenes containing anthraquinone or paraben fragments, the mechanism of their cytotoxic action has been better studied in the scientific literature.

The disadvantage of peptide bond-containing cyclophosphazenes is their low efficacy against MCF-7 breast cancer cells compared to low molecular weight compounds containing a peptide bond.

### 2.5. OCPs with Ferrocene Fragments

OCPs containing ferrocene fragments, due to the ease of changing the valence of Fe(II) and Fe(III) of ferrocene in the biological environment, can have cytotoxic action.

**Figure 12:**
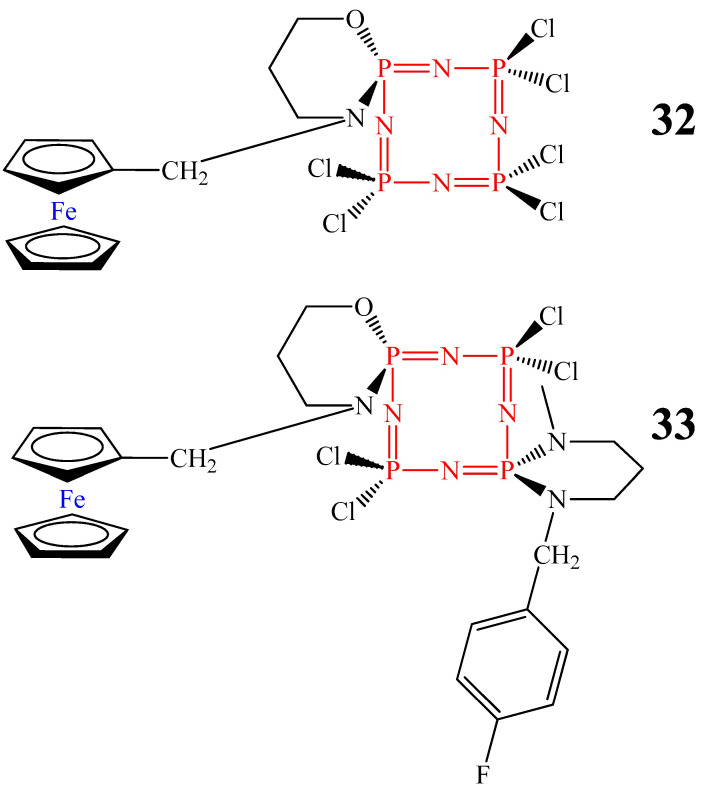
OCPs containing ferrocene fragments.

For example, monoferrocenylspirocyclotetraphosphazenes **32** and **33** (Figure 12) slowed down the proliferation of MCF7 breast cancer cells [52]. However, unlike cyclophosphazenes containing anthraquinone and paraben fragments, the presented OCPs showed antiproliferative action only at high concentrations (at least 200 μg/mL).

**Figure 13:**
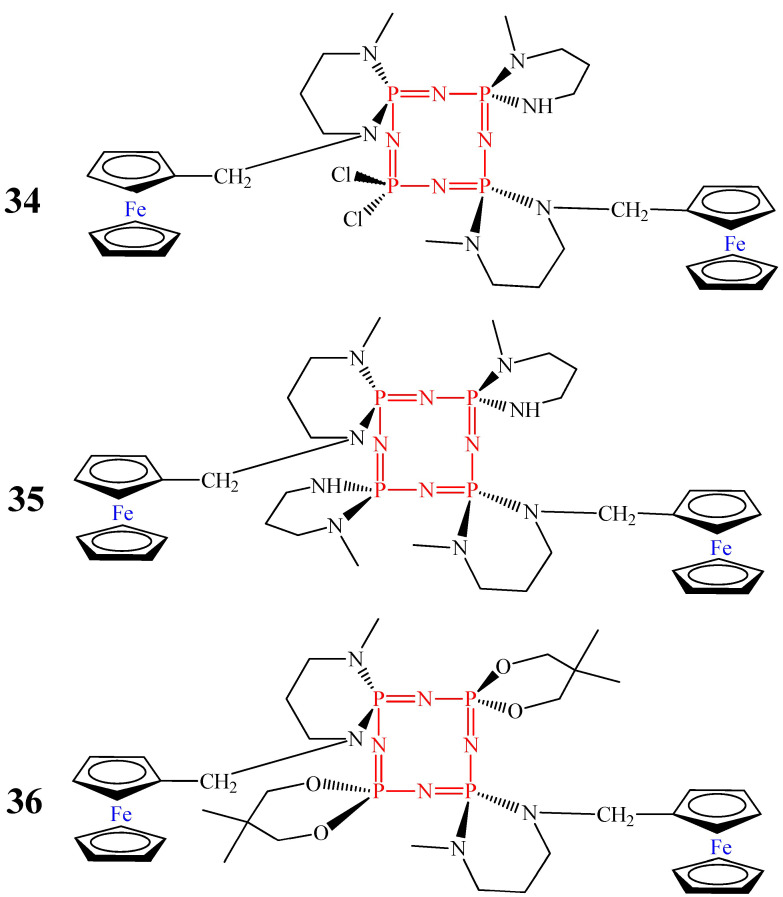
Bisferrocenylcyclotetraphosphazenes.

**Figure 14:**
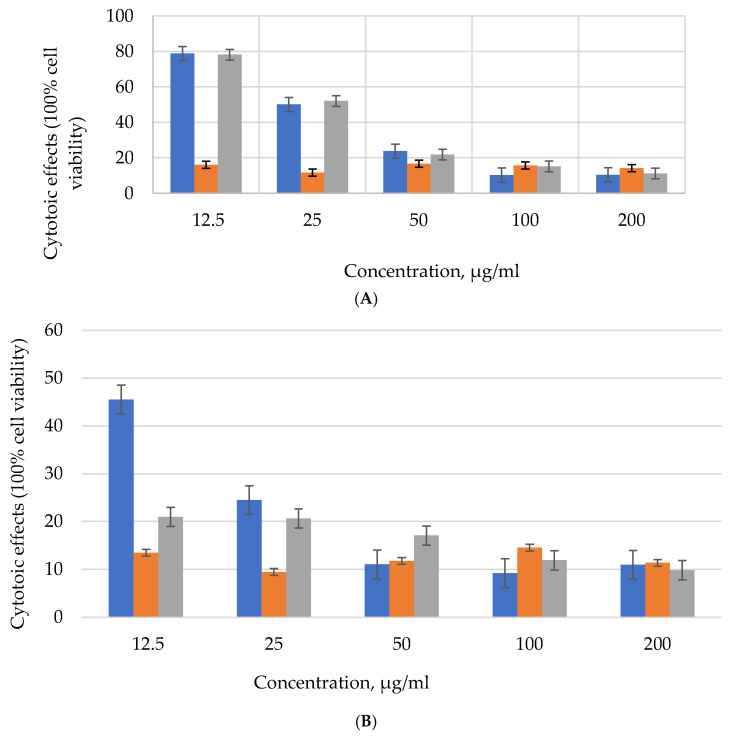
Cytotoxic effect of bisferrocenylcyclotetraphosphazenes 34–36 on DLD-1 cancer cells (**A**) and L929 mouse fibroblasts (**B**) (blue column—compound **34**, orange—**35**, and gray—**36**). Cell viability of the control group (a only medium) is 100% for DLD-1 and L929 colon cancer cell lines.

OCPs containing two ferrocene moieties (compounds **34**–**36**, Figure 13) showed cytotoxicity against DLD-1 cancer cells at lower concentrations than compounds **32** and **33** [53]. The viability of cancer cells was less than 70% at a concentration of 50 μg/mL for compounds **34** and **36**, and at a concentration of 12.5 μg/mL for compound **35** (Figure 14A). The better cytotoxic effect of compound **35**, in our opinion, is due to the presence of a larger number of aminospirocycles (four) at the phosphorus atom of phosphazene compared to compounds **34** (three rings) and **36** (two aminospirocycles).

However, compounds **34**–**36** also exhibited cytotoxic activity against non-cancerous L929 cells (Figure 14B), as did doxorubicin, which is a drawback.

**Figure 15:**
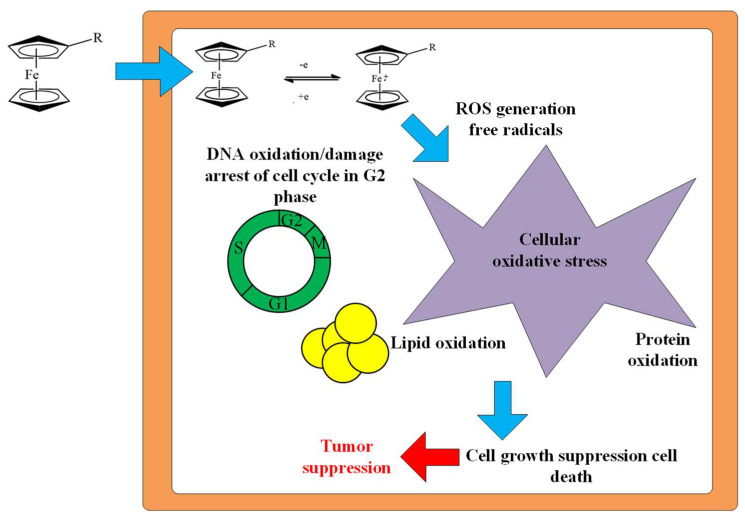
Mechanism of antitumor action of OCPs containing ferrocene fragments (R is the rest of the phosphazene molecule). The cancer cell membrane is shown in brown [54].

However, the mechanism of action of cyclophosphazenes with ferrocene fragments on cancer cells has not been studied in [52,53]. In our opinion, the mechanism of action of cyclophosphazenes containing ferrocene fragments is based on the Fenton reaction involving Fe(II) ferrocene. Ferrocene fragments allow OCPs to penetrate through the membrane of a cancer cell, and the cationic oxidized form formed under the action of oxygen in the cell ensures the solubility of OCPs in the hydrophilic environment of the cytoplasm of the cancer cell (Figure 15). As a result, during oxidation-reduction reactions in the cancer cell, ROS (superoxide anion radical O_2_^−·^ and hydroxyl radical ·OH) are formed, which provoke oxidative damage to DNA, oxidative stress, the oxidation of lipids and proteins and, ultimately, tumor suppression [54].

Thus, the assumption about the specificity of the cytotoxic action of OCPs with ferrocene fragments was not confirmed. Based on the data of works [52,53], cyclophosphazenes with ferrocenyl fragments have a cytotoxic effect not only in relation to cancer cells, but also in relation to normal L929 cells, which limits their use as PASs. In addition, they act at higher concentrations than commercial drugs, such as doxorubicin [52].

Among the cyclophosphazenes considered, the most promising, in our opinion, for use in the treatment of prostate cancer are cyclophosphazenes containing chalcone fragments, in the treatment of ovarian cancer and colon cancer—cyclophosphazenes containing peptide bonds, and in the treatment of breast cancer—cyclophosphazenes containing pyrrolidine and piperidine rings, since they have a cytotoxic effect at the lowest concentration (see Table S1 in the Supplementary Materials).

### 2.6. Proton Salts of OCPs

OCPs containing groups exhibiting base properties are capable of forming stable protic salts with inorganic and organic acids. Compared to non-solvated OCPs, protic salts are more soluble in water and physiological fluids, making them promising for use in pharmaceuticals.

In works [55,56], the proton salts of OCPs containing tetrapyrrolidine, tetrapiperidine, tetramorpholine, and tetra-1,4-dioxa-8-azaspiro [4,5]decane fragments were synthesized.

**Figure 16:**
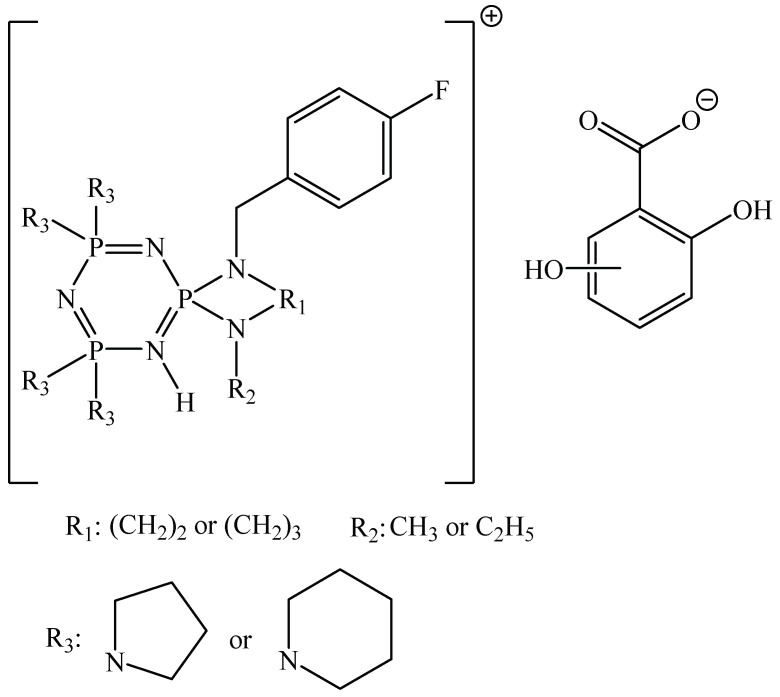
Protic salts based on OCPs with 2,5- or 2,6-dihydroxybenzoic acids [55].

The OCP salts shown in Figure 16 had better antitumor activity against A549 lung cancer cells (human alveolar basal epithelial adenocarcinoma cells) and Hep3B liver cancer cells (hepatocellular carcinoma cells) compared to cisplatin and 5-fluorouracil, as evidenced by lower IC_50_ values [55]. In addition, salts with gentisic (2,5-dihydroxybenzoic) acid fragments showed higher IC_50_ values than salts with γ-resorcylic acid fragments, and had lower antiproliferative activity.

The salts also had a cytotoxic effect on normal human amnion cells, causing necrosis and a loss of membrane integrity, which the authors attribute to the presence of a gentisic acid fragment. A γ-resorcylic acid fragment, on the contrary, reduced the cytotoxic effect of the salts on normal cells.

**Figure 17:**
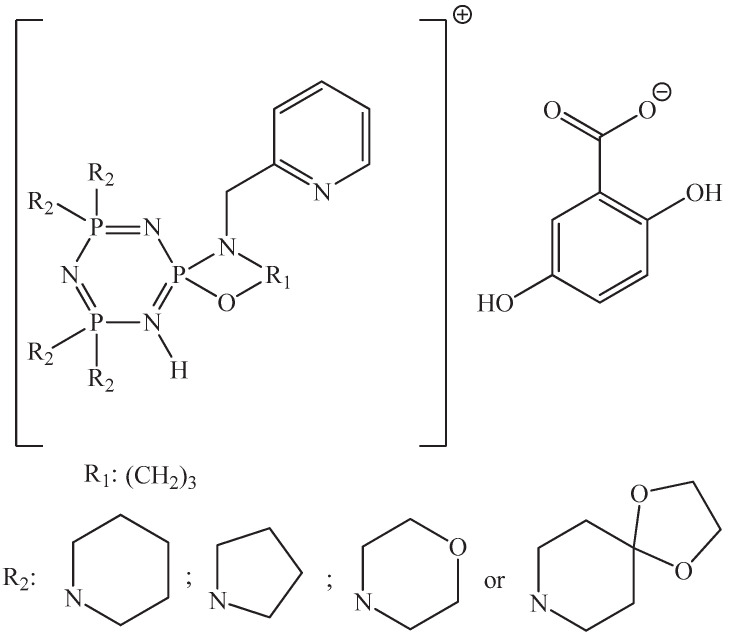
Protic salts based on 2,5-dihydroxybenzoic acid and OCPs containing tetrapiperidine, tetrapyrrolidine, tetramorpholine, and tetra-1,4-dioxa-8-azaspiro [4,5]decane fragments [56].

In work [56], it was shown that the OCP salts presented in Figure 17 have antitumor activity against MDA-MB-231 breast cancer cells, and that the greatest cytotoxic effect was observed in salts containing tetrapiperidine fragments.

The viability of mouse fibroblasts L929 was more than 80%, indicating the non-toxicity of the salts. Unfortunately, the mechanism of the antitumor action of the salts is not presented in the work.

However, from study [55] it is evident that at high salt concentrations (more than 80 μg/mL), cell apoptosis occurs, accompanied by the formation of cytoplasmic vesicles, abnormal globular structures, and apoptotic cell bodies. The authors explain that the apoptosis of cancer cells happens via the binding of proton salts of OCPs with calf thymus DNA, due to groove binding mode and electrostatic interactions with the formation of stable adducts.

It can be concluded that the general disadvantage of the considered OCPs and their salts is the complexity of their targeted delivery to the tumor. Another, more promising approach, in our opinion, is the use of cyclomatrix OCPs as carriers of PASs with antitumor action, which will be discussed below.

### 2.7. Cyclomatrix OCPs as Carriers of PASs

Microspheres and nanospheres based on cyclomatrix phosphazenes, due to the formation of appropriate functional groups on their surface, are capable of acting as carriers of PASs with antitumor activity.

**Figure 18:**
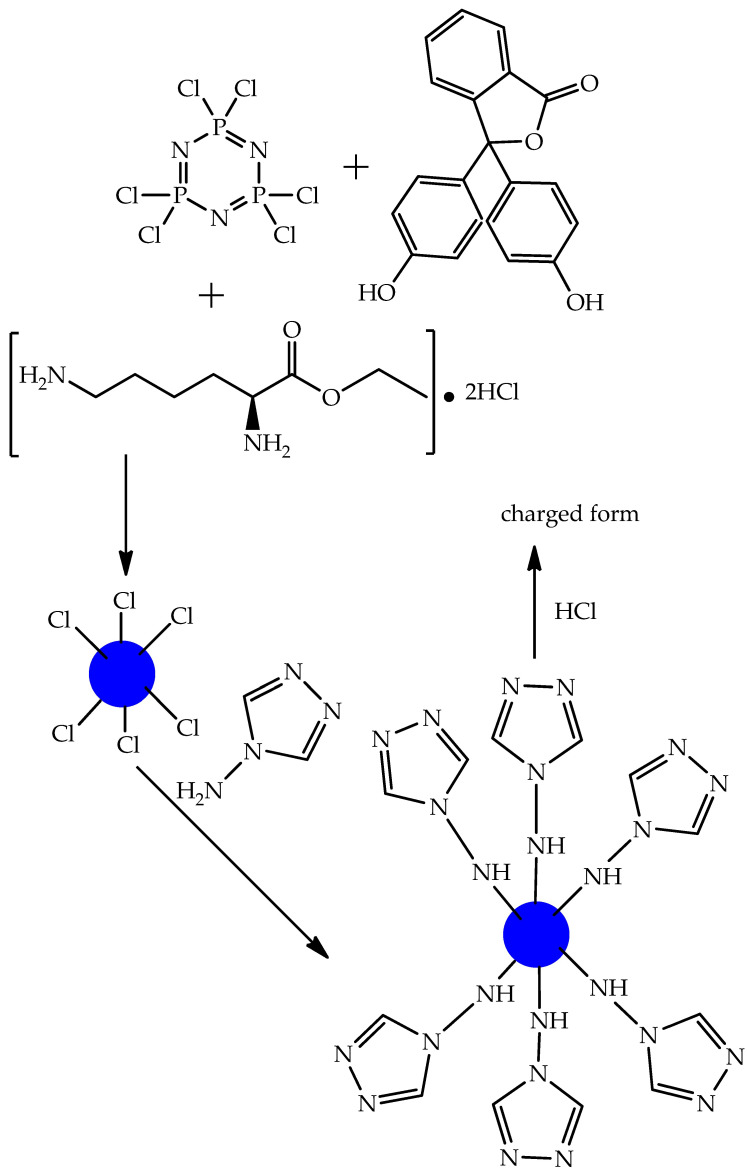
Scheme of synthesis of surface-charged microsphere.

**Figure 19:**
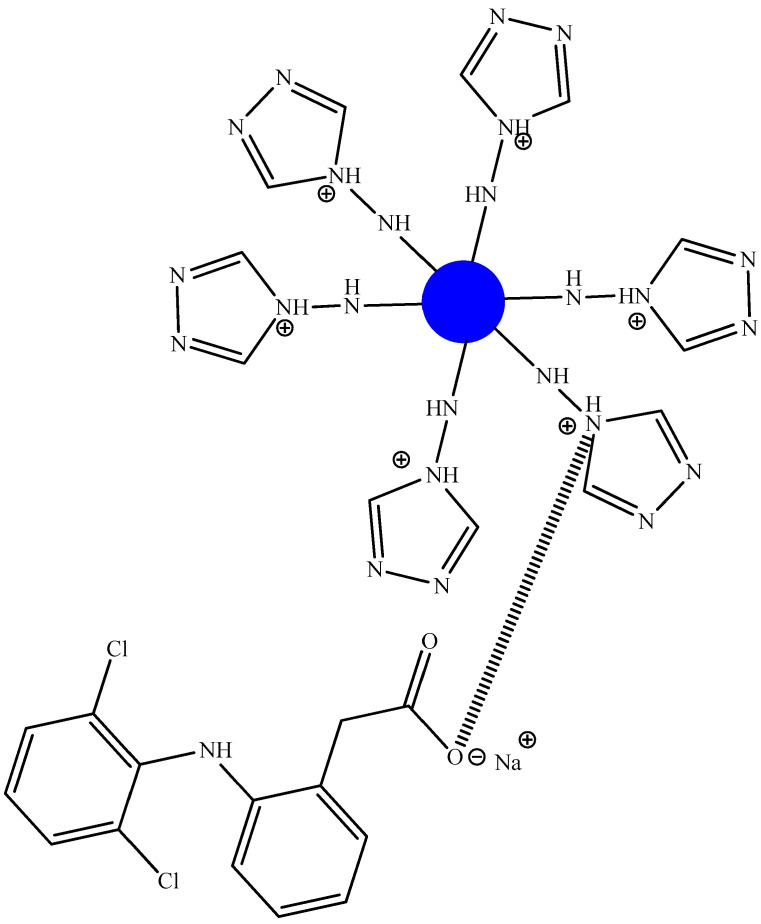
Electrostatic interactions of surface groups of OCP microspheres and sodium diclofenac.

For example, the authors of [57] found that spherical microspheres quaternized in an acidic medium (average diameter 2.5 μm) and synthesized from hexachlorocyclotriphosphazene, phenolphthalein, and dihydrochloride of ethyl ester of L-lysine and 4-amino-4H-1,2,4-triazole (Figure 18) are capable of acting as carriers of anionic PASs, such as sodium diclofenac. The authors explain this fact as being attributable to electrostatic interactions (Figure 19), contributing to the good holding capacity of the microspheres. Microspheres loaded with PASs had a cytostatic effect on HT-29 cancer cells.

However, in work [57], there is no detailed explanation of the choice of initial reagents for the synthesis of microspheres, except for their biocompatibility and biodegradability, and the formation of sodium chloride during the interaction of quaternized microspheres with sodium diclofenac is also not taken into account.

**Figure 20:**
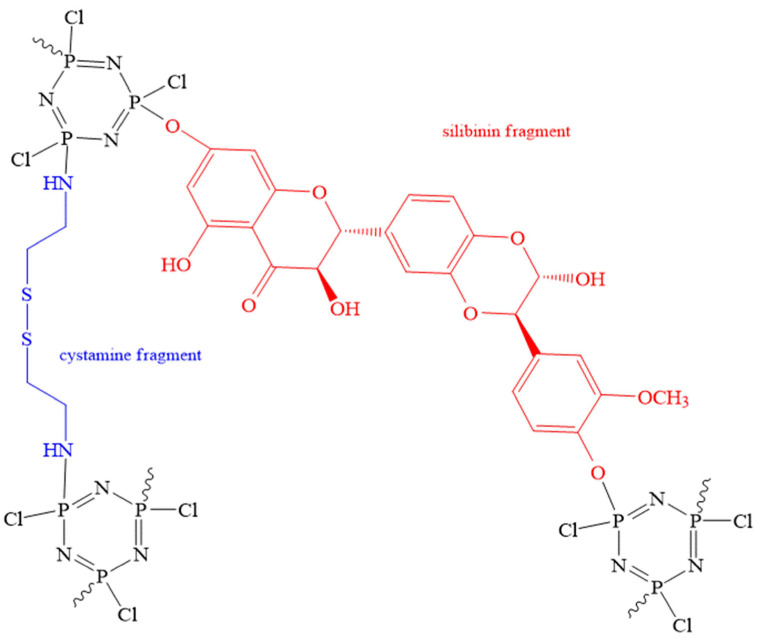
Structure of cyclomatrix OCPs used as carriers of doxorubicin.

Nanospheres, in contrast to microspheres, are more promising for the targeted delivery of PASs. This is due to the ability of particles to pass through fenestration and the vascular network [58]. Nanospheres obtained from HCP, cystamine dihydrochloride, and the cytostatic drug silibinin (Figure 20) can act, for example, as carriers of doxorubicin [59]. Their ability to be nanocarriers of cytostatics is due to their tendency toward biodegradation in tumor tissues, due to the presence of disulfide bonds active in oxidation-reduction reactions and pH-sensitive amino groups.

The nanospheres obtained in the work [59] were more effective against MDA-MB-231 breast cancer cells compared to free doxorubicin. The authors studied the release of doxorubicin from nanospheres into a phosphate buffer solution containing glutathione (a tripeptide based on glutamate, cysteine, and glycine) at different pH levels and found that at a pH of 7.4, only 16.7% of doxorubicin was released within 24 h, and in a slightly acidic medium (pH 6.5)—28.6%. In the presence of glutathione (10 mmol/L), the amount of doxorubicin released in a slightly acidic medium increased to 93.1%. The authors attribute this to the fact that the medium containing 10 mmol/L of glutathione has a pH value similar to that of tumor tissue, and that as a result of redox interaction with glutathione, the S-S disulfide bonds in the nanospheres are destroyed. In this case, glutathione is converted into glutathione disulfide.

Unfortunately, work [59] does not explain the interactions of doxorubicin with the cyclic matrix phosphazene carrier. It can be assumed that hydrogen bonds are formed between the hydroxyl groups of silybin and doxorubicin. It would also be interesting to know at what part the residual chlorine atoms at the phosphorus atoms of the phosphazene cycle, which are probably hydrolyzed, take in the biodegradation of the nanospheres.

### 2.8. OCPs in Theranostics

In the diagnosis and treatment of cancer, the visualization method is of great importance. Existing methods such as X-ray, ultrasound, computed tomography, and magnetic resonance imaging are not applicable for the detection of cancer cells and cancer cell surface markers in targeted cancer treatment [60]. A promising replacement for these methods is theranostics, which combines the diagnosis and treatment of cancer, using theranostic agents that allow for the recording of the accumulation of PASs directly in the tumor under the influence of laser radiation.

**Figure 21:**
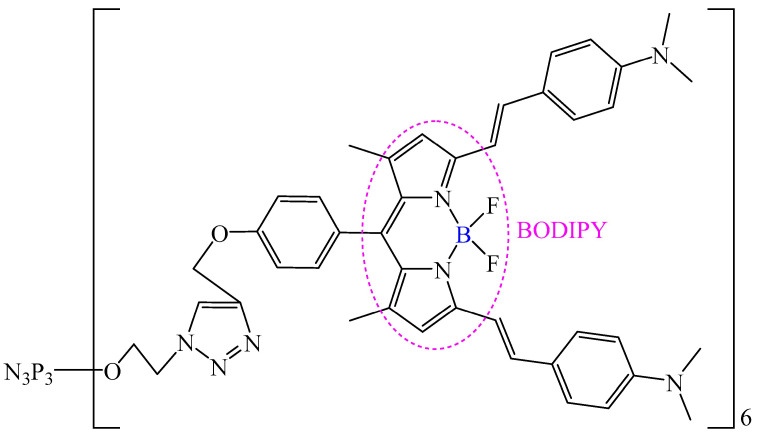
Hexa-BODIPY-containing cyclophosphazene, where P_3_N_3_ is the phosphazene ring.

Unlike phosphazene microspheres, OCP nanoparticles have a size of less than 100 nm and can be used in the field of theranostics [61]. For example, BODIPY-OCP (Figure 21) exhibited high photothermal activity and phototoxicity towards breast cancer cells (cell line 4T1) due to the generation of singlet oxygen in cancer cells, leading to their apoptosis. Unfortunately, the mechanism of singlet oxygen generation involving BODIPY-OCP is not presented in [61], which makes it difficult to understand its action as a photosensitizer. Furthermore, it is unclear whether phototoxicity is actually due to the effect of radiation on phosphazene.

BODIPY-OCP nanoparticles were found to exhibit near-infrared fluorescence in the cytoplasm of cancer cells. In vivo experiments in mice demonstrated that the nanoparticles provided visual control of tumor growth inhibition, as evidenced by the distinct fluorescence at the tumor site after 24 h. The subcutaneous injection of 4T1 cancer cells into mice and treatment with phosphazene and laser (655 nm wavelength) showed the disappearance of a tumor with a volume of 100 mm^3^.

The advantage of BODIPY-OCP compared to other photosensitizers, such as simple 4,4-difluoro-4-bora-3a,4a-diaza-s-indacene (BODIPY), is the minimal adverse effect on normal tissues, the retention of fluorescence after encapsulation in aqueous solution, as well as the accumulation in the tumor for a long time due to the nanosize (approximately 93 nm) and the enhanced permeability and retention (EPR) effect. The disadvantage of BODIPY-OCP is the need for its encapsulation with an amphiphilic polymer (e.g., polyethylene glycol-polylactic acid copolymer PEG5000-PLA3000) to increase its solubility in water, impart biocompatibility and stability in the vascular bed, and conduct in vivo testing. However, it is known that PEG can be oxidized in a physiological environment to form toxic products containing aldehyde groups, and is also immunogenic and antigenic [62].

According to the analysis of the reviewed works, it is necessary to highlight the main studies that are important for understanding the mechanisms of action of phosphazenes on cancer cells of various morphologies and bringing valuable information for modeling new compounds with antitumor action. However, the number of studies that consider the mechanism of action is limited. Studies were mainly devoted to the study of cytotoxic activity. Thus, in 14 articles, studies of in vitro cytotoxic activity with colorimetric MTT and WST assays were carried out, using tetrazolium salts—3-(4,5-dimethylthiazol-2-yl)-2,5-diphenyltetrazolium bromide in the MTT assay and 1-(4-[3-(4-iodophenyl)-2-(4-nitrophenyl)-2H-5-tetrazolio]-1,3-benzenesulfonate) in the WST assay. Four articles studied the interactions of phosphazenes with cancer cell DNA via molecular docking studies and the comet assay, two articles studied antimigratory activities via in vitro scratch analysis, one article studied tumor cell apoptosis, and one article studied cell morphology. The prevalence of the MTT and WST methods is probably due to their simplicity, low cost, safety, and high sensitivity. In turn, the study of the interaction of phosphazenes with enzymes and cancer cell DNA requires special software, such as the AutoDock Vina program.

It can be concluded that the works reviewed do not contain enough research to implement them in clinical practice. Further pharmacokinetic studies are needed, including constructing a release profile of OCPs in fluids simulating human physiological fluids (gastric fluid pH 1.2, intestinal fluid pH 6.8, and colon fluid pH 7.4), stability studies in accordance with the International Council for Harmonization of Technical Requirements for Pharmaceuticals for Human Use (ICH) (2003) code Q1A(R2) (stability testing of new medicinal substances and products), and in vivo studies, such as acute toxicity studies in zebrafish.

Therefore, for use in clinical practice, OCPs, depending on the method of their use, are more promising as antimicrobial drugs, since they are subject to fewer requirements compared to cytostatics. This is due to the fact that, unlike antimicrobial drugs, cytostatics often cause serious complications in patients receiving treatment.

## 3. OCPs with Antimicrobial Action

The problem of microorganism resistance to currently used PASs, as well as the side effects of PASs (for quinolones and sulfonamides—fever and rash, for aminoglycosides—nephrotoxicity, and macrolides—nausea, vomiting, abdominal discomfort, and diarrhea) require the development of alternative drugs. Phosphazenes are promising candidates for the treatment of infectious diseases. It is assumed that phosphazenes will have a minimum number of side effects compared to antibiotics, since the biodegradation of phosphazenes releases harmless ammonium dihydrophosphate that is non-toxic to tissues.

### 3.1. OCPs with Antituberculosis Action

Tuberculosis caused by *Mycobacterium tuberculosis* is one of the leading causes of death according to the World Health Organization. Due to the adaptation mechanisms of mycobacteria, they develop resistance even to very strong anti-tuberculosis drugs, such as rifampicin and isoniazid [63]. In turn, second-line antituberculosis drugs (bedaquiline and delamanid) cause many side effects, one of which is the prolongation of the QT interval on the electrocardiogram [64]. Therefore, it is necessary to develop PASs with a minimum number of side effects.

Among OCPs, phosphazenes containing ferrocenyl, pyrrolidine, morpholine heterocycles, and DASD cycles have anti-tuberculosis activity.

**Figure 22:**
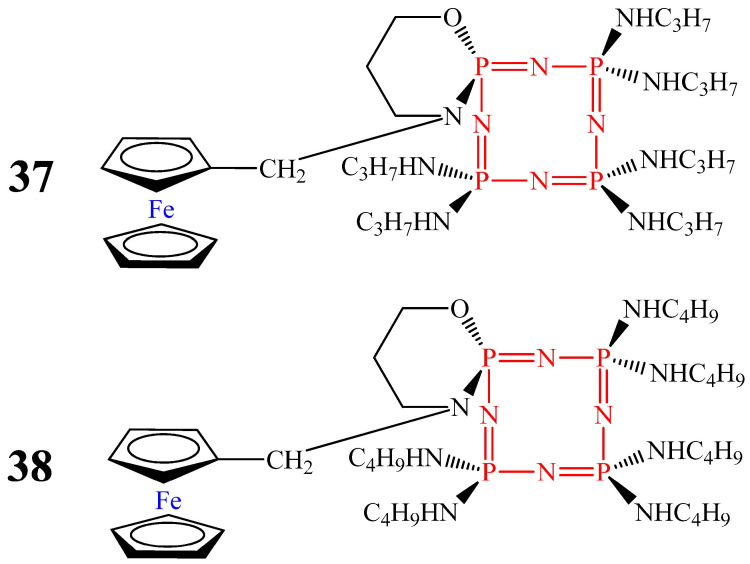
Monoferrocenyl spirocyclotetraphosphazenes.

In [65], it was shown that monoferrocenylhexaamino(N/O) spirocyclotetraphosphazenes (compounds **37** and **38**, Figure 22) and compound **32** have activity against *Mycobacterium tuberculosis* (strain H37Rv). The minimum inhibitory concentration (MIC) for compound **37** (35 μg/mL) was two times lower compared to those of compound **38** (70 μg/mL) and compound **32** (80 μg/mL) [65]. However, the MIC values were higher than those of the drugs currently used against *Mycobacterium tuberculosis*—rifampicin (1.0 μg/mL), isoniazid (1.0 μg/mL), ethambutol (10 μg/mL), and streptomycin (10 μg/mL), which is a disadvantage of OCPs **32**, **37**, and **38**.

**Figure 23:**
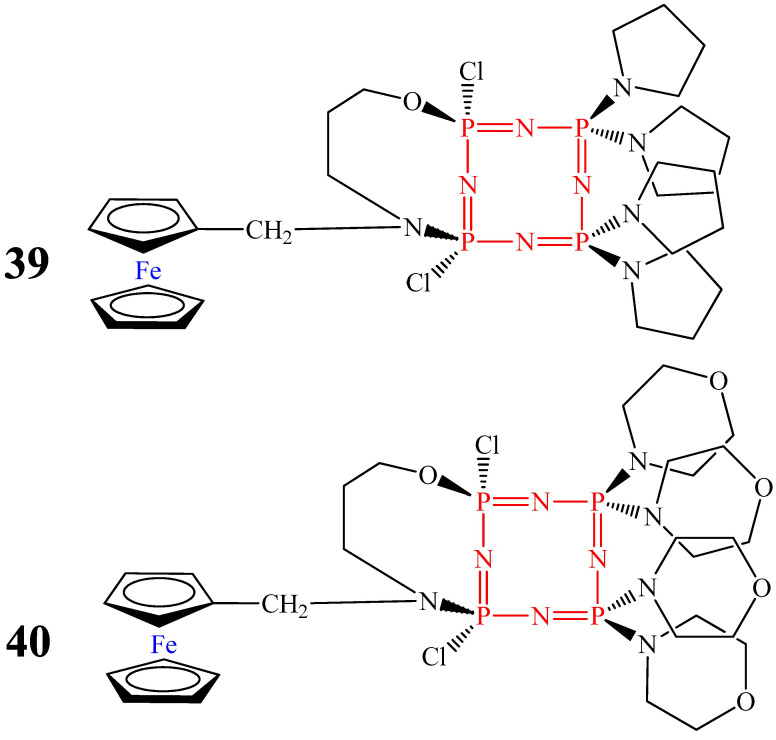
Monoferrocenyl ansacyclotetraphosphazenes containing pyrrolidine and morpholine heterocycles.

Compared with OCPs **32** and **38**, compounds **35** and **36** containing two ferrocenyl moieties and diaminospirocycles [53], as well as monoferrocenylhexaamino(N/O) ansacyclotetraphosphazenes containing pyrrolidine and morpholine rings (compounds **39** and **40**, Figure 23), showed lower MIC values (less than 40 μg/mL). For OCPs **35** and **36**, the MIC values were 30 μg/mL and 16 μg/mL, respectively, and for OCPs **39** and **40**, the MIC values were 38 μg/mL and 36 μg/mL, respectively [66]. The lower values of compounds **35** and **36**, in our opinion, is due to the presence of two ferrocenyl fragments in their composition, and the lower MIC value of compound **40** is due to the presence in the structure of the molecule of a morpholine ring, which has antimycobacterial effects along with ferrocene [67].

**Figure 24:**
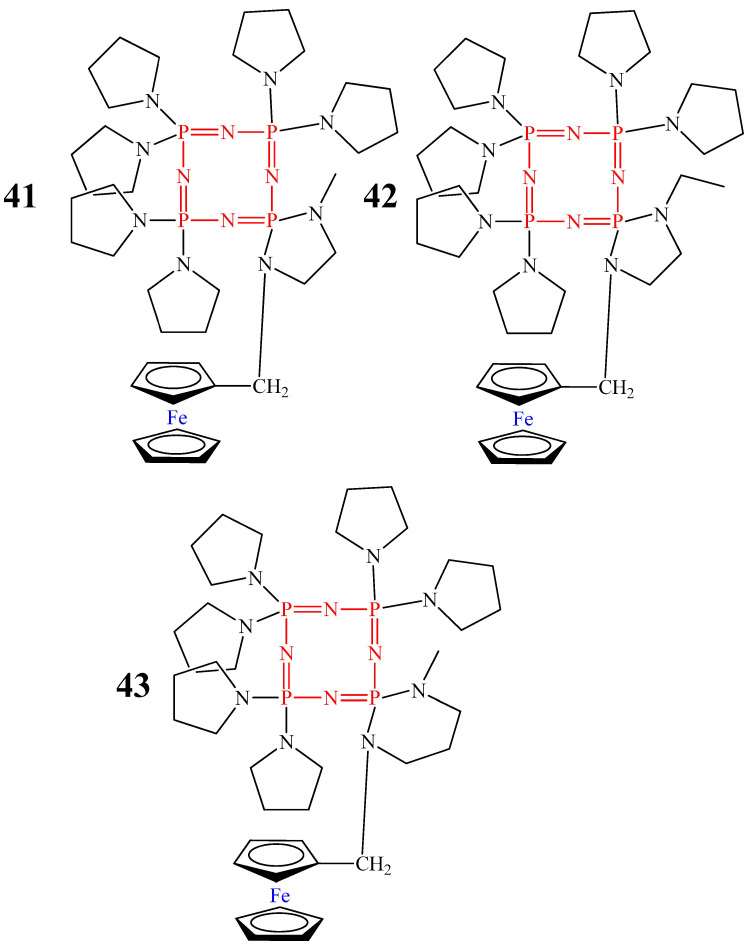
Monoferrocenylcyclophosphazenes with pyrrolidine rings.

In contrast to compounds **39** and **40**, OCPs **41**–**43** (Figure 24) showed MIC values of 3 μg/mL, close to the commercial antibiotics rifampicin (1.0 μg/mL) and isoniazid (1.0 μg/mL) [68]. This is probably due to the presence of six pyrrolidine heterocycles and one diaminospirocycle in their composition.

Thus, the most promising compounds for the treatment of tuberculosis are cyclophosphazenes containing ferrocene and several pyrrolidine rings (Table S2, Supplementary Materials), since they have the lowest MIC values. Despite the potential non-toxicity of the compounds considered, their disadvantage is the formation of geometric (non-geminal, geminal, and cis/trans) and chiral isomers during their synthesis, which are difficult to separate. In addition, the works considered do not describe the mechanism of the anti-tuberculosis action of cyclophosphazenes containing ferrocene.

### 3.2. OCPs with Antibacterial and Antifungal Action

In addition to anti-tuberculosis action, cyclophosphazenes containing N-heterocycles, ferrocene, or fluorine atoms have antibacterial and antifungal action against Gram-positive (*S. aureus*, *B. cereus*, *B. subtilis*, and *E.* faecalis, etc.), Gram-negative bacteria (*P. vulgaris* and *K.* pneumaniae, etc.), and fungi of the genus *Candida*.

For example, cyclophosphazene **43**, containing pyrrolidine heterocycles and ferrocene, showed antimicrobial action against Gram-positive bacteria *B. cereus*, *B. subtilis*, and *E. faecalis* and fungi *C. albicans*, *C. krusei*, and *C. tropicalis*, with a MIC and minimum fungicidal concentration (MFC) value of 19.5 μmol/L [68]. These MIC and MFC values were lower than those of the commercial antibiotics chloramphenicol and ketoconazole in terms of antibacterial and fungicidal activity, respectively (Table 2). However, unlike the antibiotic chloramphenicol, OCP **43** did not have antibacterial activity against the Gram-negative bacteria *E. coli*, *P. aeruginosa*, *S. typhimurium*, or *K. pneumoniae*. Unfortunately, there is no explanation for this fact in the work [68]. Probably, this is due to the difference in the structure of the cell wall of Gram-positive and Gram-negative bacteria. It is easier for cyclophosphazene molecules to penetrate the porous outer layer of the peptide glycan of Gram-positive bacteria and destroy it compared to the imperforate outer membrane of Gram-negative bacteria [69].

Compared with cyclophosphazene **43**, OCPs **44** and **45** showed antibacterial activity against both Gram-positive and Gram-negative bacteria. In particular, compound **44** had antibacterial activity against *B. subtilis* (+), *P. vulgaris* (−), and *K. pneumoniae* (−) [52], and compound **45** against *E. faecalis* (+), *S. aureus* (+), *B. cereus* (+), and *K. pneumoniae* (−) [70]. This is probably due to the presence of fluorine atoms in their composition, which improves penetration through the outer membrane of Gram-negative bacteria by increasing the lipophilicity of the molecule.

OCPs **46**–**48** had antibacterial activity against the bacteria *E. faecalis* (+), *S. aureus* (+), *B. subtilis* (+), *B. cereus* (+), *K. pneumoniae* (−), and *P. vulgaris* (−); however, at higher MBC values (312.5–2500 μmol/L) [71] than compounds **44** and **45**, which limits their use as antimicrobial PASs.

A disadvantage of the compounds considered is their insolubility in water, which may limit their use as antibiotics.

Unlike the compounds **43**–**48** considered above, OCPs containing diethanolamine fragments and chlorine atoms in phosphazene cycles (compounds **49**–**55**) are water-soluble antimicrobial PASs.

In work [72], the antimicrobial action of compounds **49**–**55** against Gram-negative bacteria *E. coli*, Gram-positive bacteria *S. aureus*, and fungi *C. albicans* was investigated. The authors of work [72] showed that the lowest MIC values (9–45 μg/mL) against the indicated microorganisms were observed for compound **55**, and the highest for compounds **52** and **53** (more than 1000 μg/mL). The MIC values of compound **55** were close to the MIC values of the antibiotic kanamycin.

The same research group obtained a water-soluble OCP with an N-(1-naphthyl)ethylenediamine fragment, compound **56**, which has an antimicrobial effect against *E. coli*, *S. aureus* bacteria, and *C. albicans* fungi, similarly to compound **55** (MIC values 4–8 μg/mL) [73].

**Figure 25:**
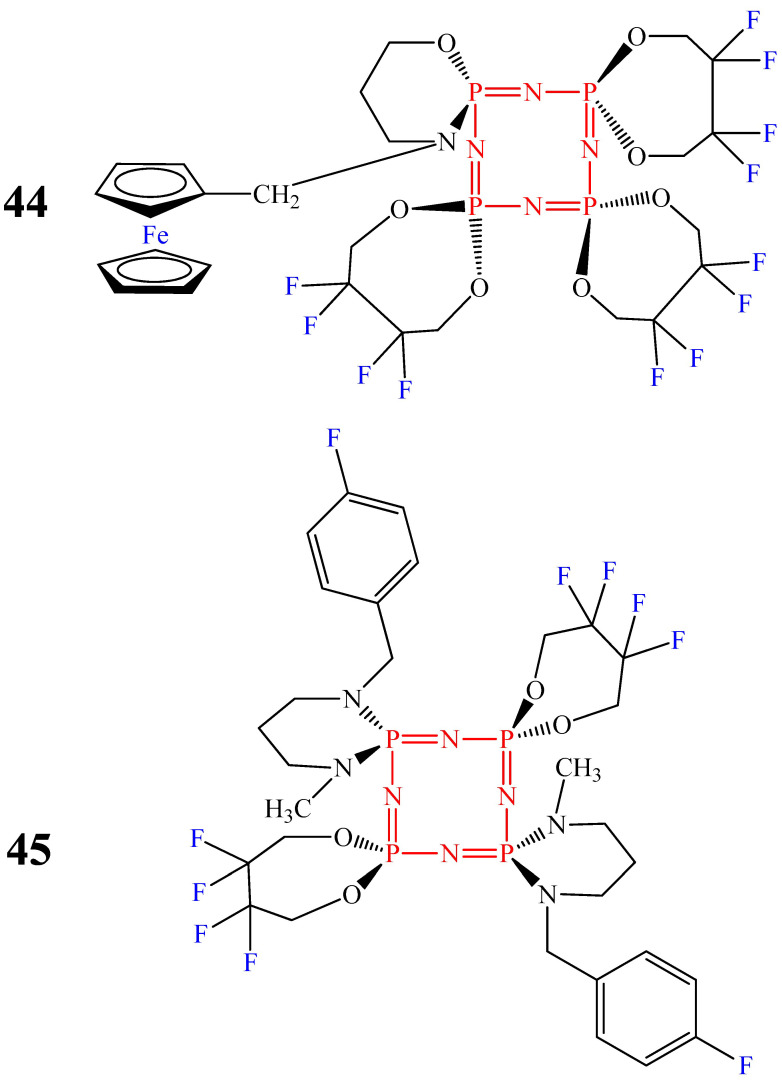

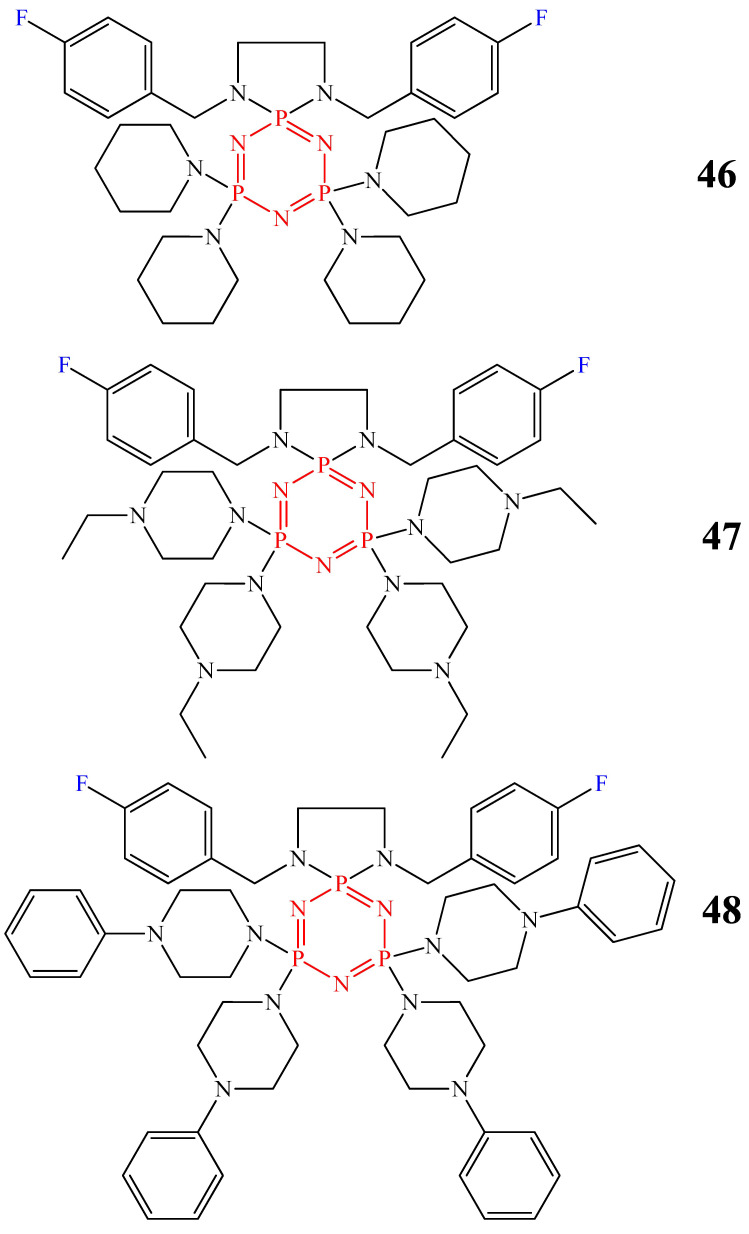

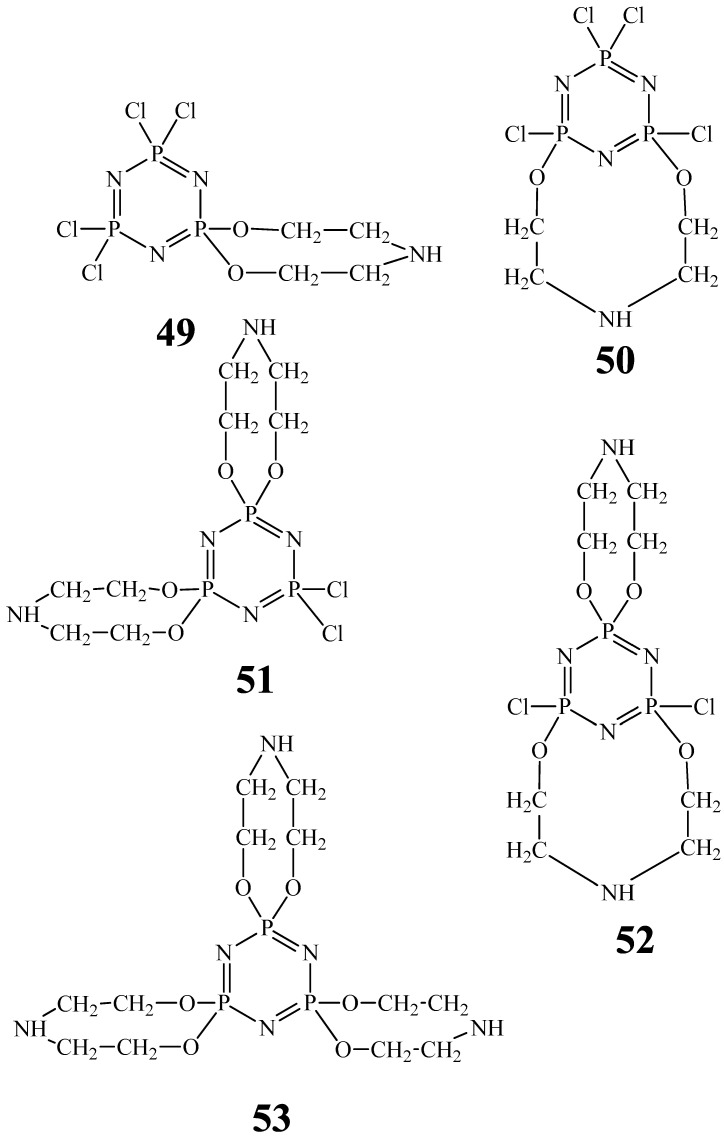
Cyclophosphazenes with fluorine or chlorine atoms.

**Figure 26:**
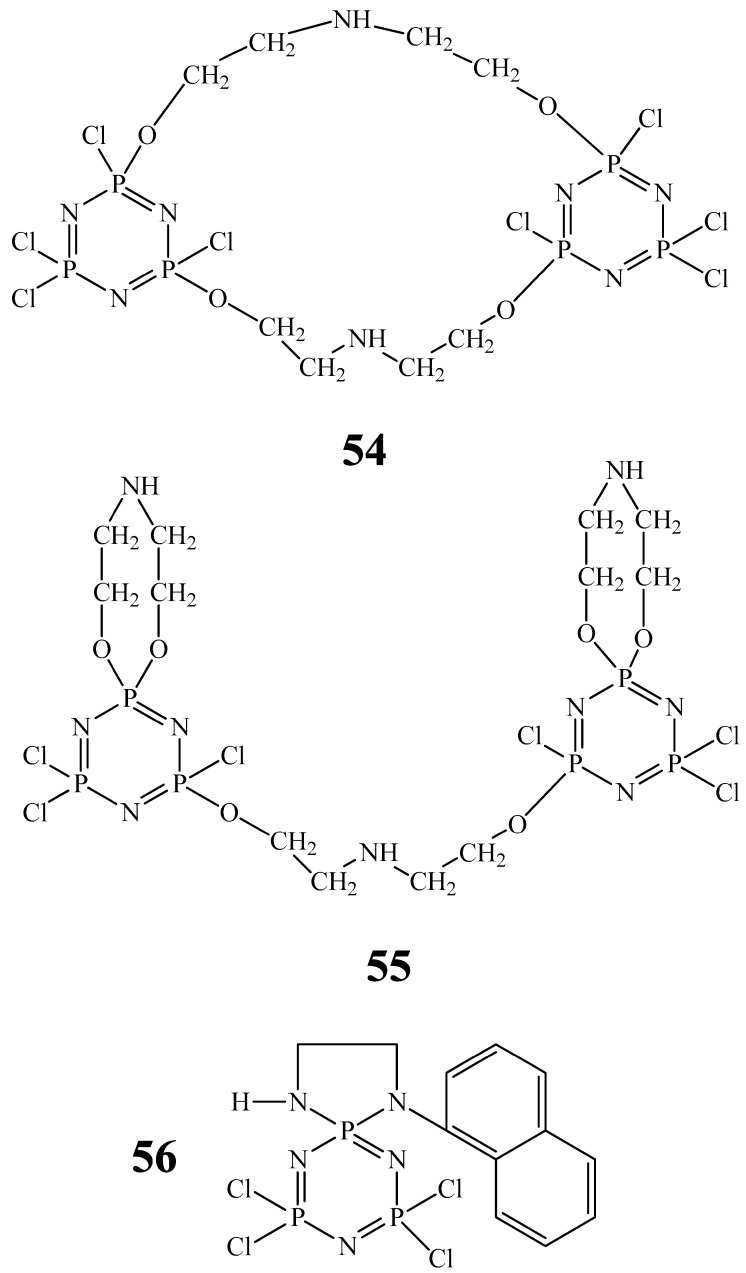
Cyclophosphazenes with chlorine atoms.

Unfortunately, the works [72,73] do not explain the differences in the MIC value depending on the molecular architecture of the synthesized OCPs, nor on the mechanism of their antibacterial action. In addition, compounds **49**–**56** (Figure 25 and Figure 26) are hydrolytically unstable due to the presence of free chlorine atoms, which complicates their storage and limits their use in pharmaceuticals.

The antibacterial action of phosphazenes containing amino groups is probably due to the nucleophilicity of nitrogen atoms, leading to electrostatic interaction with the negatively charged membrane of the bacterial cell, which leads to the depolarization of the membrane, disruption of its integrity, and inhibition of energy transport and metabolism of the bacteria [74]. In addition, the antimicrobial effect can be achieved via the action of OCP degradation products.

Thus, cyclophosphazenes containing ferrocene fragments and pyrrolidine rings are promising antimicrobial agents for the treatment of candidiasis caused by fungi of the genus *Candida* and food poisoning caused by soil bacteria *B. cereus* and *B. subtilis*; cyclophosphazenes containing fluorine atoms for the treatment of urinary tract infections and urogenital infections caused by *P. vulgaris* and *K. pneumaniae*; and cyclophosphazenes with diamine spirocycles for the treatment of nosocomial infections (caused by *S. aureus*). In contrast to the anti-tuberculosis action of OCPs, the mechanism of antibacterial action of cyclophosphazenes **43**–**48** was studied on a plasmid circular double-stranded bacterial DNA molecule via electrophoresis in agarose gel, and it was shown that cyclophosphazenes bind to the G/G and A/A nucleotides of the pBR322 plasmid DNA, which leads to the transformation of the supercoiled form and the single circular form into the linear DNA form [52,68,70,71]. However, it is necessary to create OCPs that combine solubility in water and low MIC, MBC, or MFC values, as well as in vivo studies.

Nevertheless, to prevent the “explosive” release of PASs into physiological environments and ensure their prolonged and controlled action, the possibility of not only oral, but also transdermal delivery of PASs is a promising area of research in the development of hydrogels as PAS carriers.

## 4. OCPs in Gels

OCPs containing six aldehyde groups in aromatic radicals can be used in biomedicine and pharmaceuticals as cross-linking agents for biocompatible polymers such as chitosan [75,76] and polyvinyl alcohol [77], etc. Hydrogels obtained on the basis of biocompatible polymers cross-linked with OCPs can act as carriers of PASs. The advantage of OCPs compared to ionic cross-linking agents (phosphates and sulfates) is the formation of a stable spatial network. In addition, the higher functionality of cyclophosphazenes increases the cross-linking density of the polymer network.

**Figure 27:**
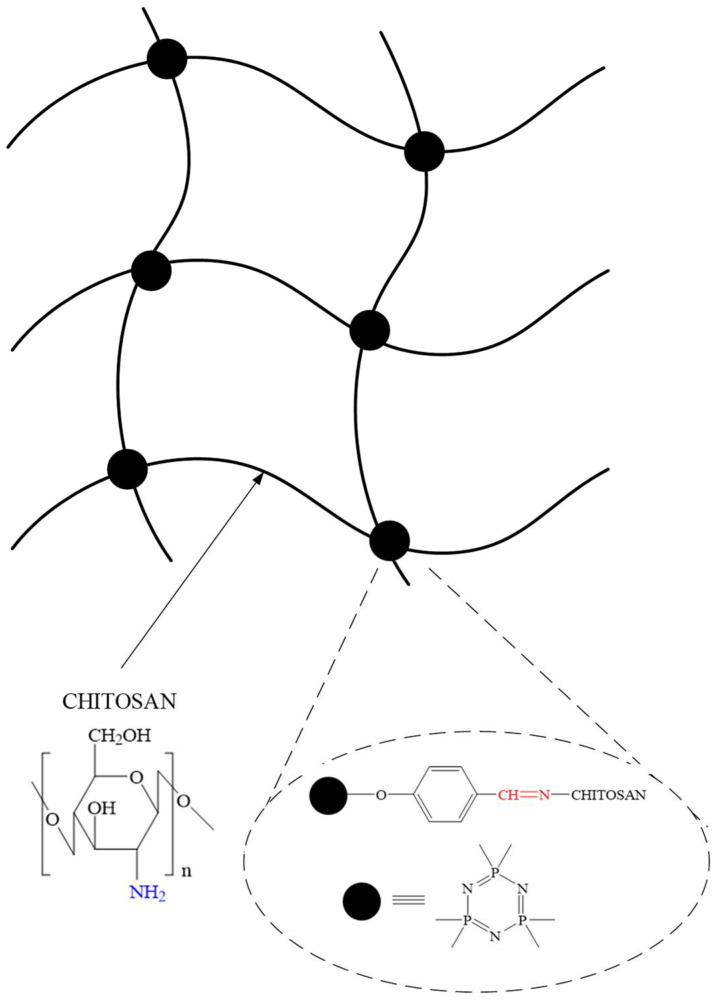
Chitosan hydrogel network.

In particular, in [75], a gel based on chitosan cross-linked with hexakis(4-formylphenoxy)cyclotriphosphazene (FPP) was obtained. This gel acted as a carrier of the antibiotic amoxicillin. The cross-linking reaction of chitosan with FPP took place at a temperature of 35 °C using non-toxic DMSO to dissolve FPP and acetic acid to dissolve chitosan. FPP formed a network polymer structure from chitosan chains during the reaction of the aldehyde groups of FPP with the amino groups of chitosan (Figure 27).

**Figure 28:**
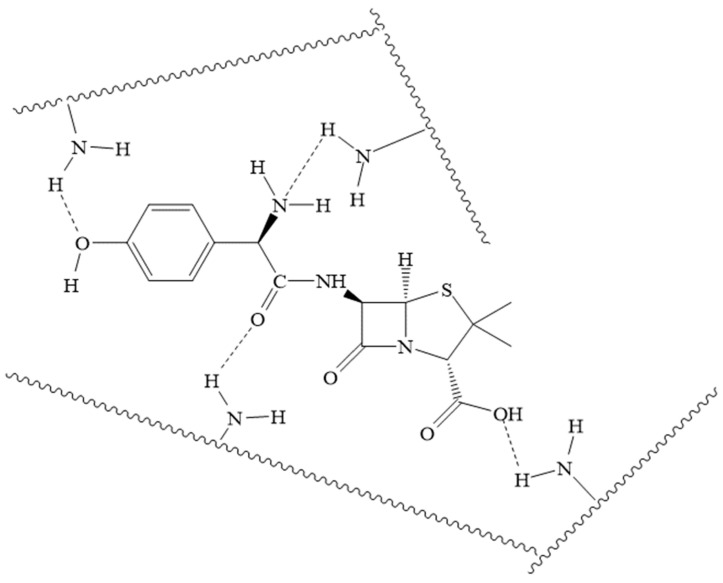
Probable hydrogen bonds of amoxicillin with chitosan chains of the gel.

The resulting hydrogel exhibited a high swelling capacity in water (around 4000%) and was sensitive to pH changes, allowing it to degrade under various physiological conditions. In a simulated gastric fluid environment (pH 1.1), the azomethine groups were rapidly hydrolyzed, causing biodegradation and the explosive release of the antibiotic bound to the gel matrix via hydrogen bonds (Figure 28) and van der Waals interactions within the first 15 min.

However, the rapid release of the PAS is undesirable, so the authors investigated the release of the antibiotic in other media and found that in a phosphate buffer solution (pH 7.0) and simulated body fluid (pH 7.4) simulating the intestinal environment and blood plasma, the release of the drug is not “explosive”. Unfortunately, work [75] does not provide an explanation for this fact. Probably, this is due to the slow biodegradation of the phosphazene cross-linking agent in a neutral and slightly alkaline environment, which allows for the controlled release of the antibiotic.

**Figure 29:**
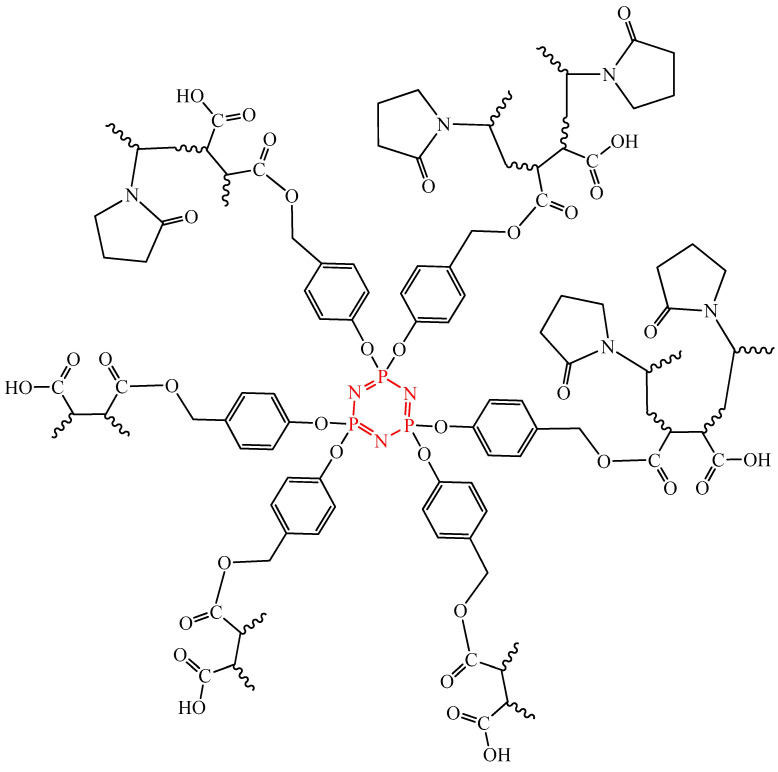
Copolymer based on cyclophosphazene containing maleic groups and N-vinylpyrrolidone.

OCPs can act not only as cross-linking agents, but also as matrices for obtaining gels for the controlled delivery of PASs. In particular, a gel based on a copolymer of OCPs with maleic groups and N-vinylpyrrolidone (Figure 29) also turned out to be sensitive to the pH of the medium [78]. At a pH of 7.4, simulating the environment in the intestine, swelling of the gel was observed, while at a pH of 2, simulating conditions in the stomach, compression of the gel was observed. The authors of the work explain this as attributable to the destruction of hydrogen bonds at pH = 7.4, creating a greater swelling force, while in an acidic medium, on the contrary, the forces of intermolecular interaction increased.

A study of the release of the antitumor drug 5-fluorouracil from a gel matrix showed that at a pH of 7.4, 54.6% of the drug is released within an hour, while at a pH of 2, only 13.9% is released, which, according to the authors of the work [78], is due to the compression of the gel in an acidic environment, which hinders the transport of 5-fluorouracil. It is worth noting that the release of the PAS from the gel matrix turned out to be “explosive”, which is a disadvantage of the gel. For a smoother release of the FAV, the authors proposed regulating the ratio of monomers, in particular increasing the content of phosphazene, which will lead to an increase in the cross-linking density and slow down the release.

Unfortunately, the authors of the work did not study the interaction of the gel matrix with the functional groups of the PAS, which is important for understanding the mechanism of drug binding, as well as the cytotoxicity of the gel in relation to fibroblasts, and degradation in vivo. In addition to the controlled delivery of PASs, gels based on natural polymers cross-linked with OCPs can be used to manufacture wound dressings [76].

For example, a gel based on sodium alginate and chitosan cross-linked with FPP showed activity against Gram-positive bacteria (*S. aureus*). However, this effect was weak, since the diameter of the inhibition zone was only 10 mm. An increase in the diameter of the inhibition zone to 30 mm was achieved by introducing copper (II) into the gel structure, which was coordinated via the nitrogen atoms of the azomethine bond and the oxygen atoms of the hydroxyl group of chitosan.

Unfortunately, OCP-based hydrogels in biomedical applications are limited to drug delivery. In turn, cyclomatrix-type phosphazenes can also be used in biosensors.

## 5. OCPs in Biosensors

For the diagnostics of heavy metal ion poisoning, as well as the monitoring and visualization of neurotransmitters for the treatment of neurodegenerative diseases in living cells, it is necessary to develop highly sensitive optical fluorescent sensors and electrochemical sensors. In order to improve the optical properties of fluorescent sensor materials, OCPs can be used, since they are an optically inert platform in the UV and visible light region. The functionalization of phosphazenes with fluorophore groups leads to the creation of a multinuclear platform with a high efficiency of intra- and intermolecular CH–π and π–π interactions [79]. To impart stability to phosphazene fluorophores upon contact with the human body and to enhance interactions with analytes, cyclomatrix phosphazene microspheres are used, since they have high surface energy, developed surface, and a large number of binding sites for ions and molecules [80].

Cyclomatrix phosphazene microspheres are obtained from various functional OCPs. For example, OCP-based microspheres containing sulfur in organic radicals to increase the electron transport capacity were synthesized from HCP and 1,4-dithiane-2,5-diol.

**Figure 30:**
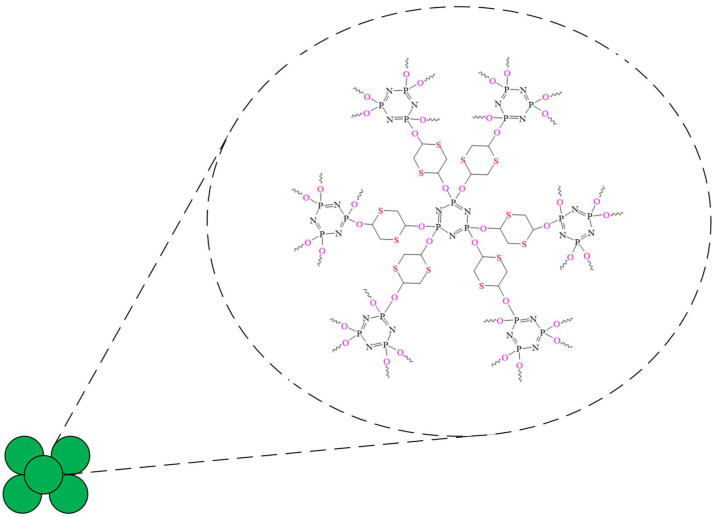
Cyclomatric phosphazene microspheres.

The microspheres have a high electron transport capacity, conductivity, and hydrophilicity, which makes them promising compounds for the manufacture of neurotransmitter sensors, in particular dopamine [81]. Unlike a non-enzymatic electrochemical sensor, which detects only dopamine, microspheres can also detect other electroactive substances, such as ascorbic and uric acids and glucose. According to the authors of [81], a sensor based on carbonized spherical microspheres (Figure 30) will have high selectivity and sensitivity even in the presence of interference.

However, further research using cerebrospinal fluid and plasma in mice is needed to apply the sensor for monitoring dopamine levels in diagnosing neurological diseases such as Parkinson’s disease and schizophrenia.

OCPs can also act as a core for obtaining ligands capable of detecting heavy metal ions in biological fluids (e.g., blood samples), high concentrations of which cause neurogenerative diseases such as Parkinson’s disease and Alzheimer’s disease [82].

**Figure 31:**
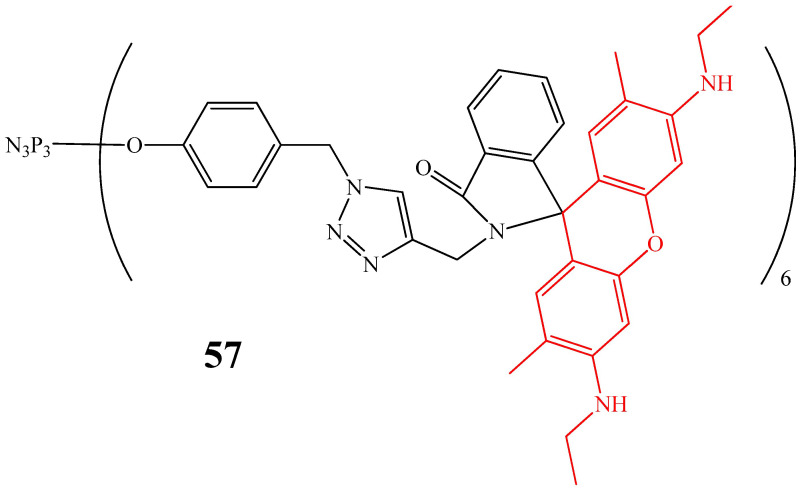
Structure of cyclophosphazene containing fragments of rhodamine 6G (highlighted in red) with a lactam ring.

OCPs capable of forming metal complexes can be used for blood analysis. For example, optical sensors based on OCPs containing rhodamine 6G (compound **57**, Figure 31), absorbing in the 532 nm region characteristic of trivalent iron ions Fe^3+^, and not absorbing light in other regions, have selectivity, a lower cost compared to instrumental methods such as atomic absorption spectroscopy [83], and can also be reused after ligand reduction using ethylenediamine [84].

It should be noted that cyclophosphazene **57** was tested using aqueous solutions, with the addition of 4-(2-hydroxyethyl)-1-piperazineethanesulfonic acid as a buffer (pH 7.4), simulating the buffer system of blood. However, due to the complex composition of blood, containing both inorganic and organic components, additional studies of the chemical sensor on whole blood samples are needed. In addition, azides were used in the synthesis of OCPs, which are potentially explosive compounds, which complicates the industrial synthesis of phosphazenes for the manufacturing of sensors.

**Figure 32:**
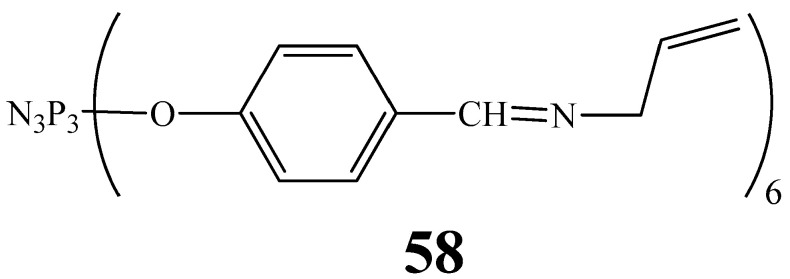
Structure of cyclophosphazene containing azomethine groups.

In addition to neurotransmitter and heavy metal ion sensors, OCPs can be used to manufacture non-enzymatic glucose sensors. For example, cyclophosphazene **58** (Figure 32) obtained in [85], which contains azomethine groups and multiple bonds, is a promising compound for the synthesis of metal-containing redox-active polymers capable of non-enzymatically oxidizing glucose on the electrode surface and possessing reversibility of electrochemical behavior, resistance to electrolyte solutions, and high redox activity due to the polydentate nature of OCPs. However, there are no studies in the scientific literature on the mechanism of action of a redox-active metal-containing polymer based on compound **58**.

Despite the high performance of the OCP-based biosensors presented in this section, additional studies using physiological fluids and laboratory animals are required for their use as sensors of biologically active substances and heavy metals.

Compared to the above-mentioned areas of application, restorative dental materials are not subject to mandatory studies involving laboratory animals and clinical trials [86]. Therefore, another important area of OCP use is dentistry.

## 6. OCPs in Dentistry

In the field of dentistry, there is a need to develop modifiers that would give dental filling material high adhesive strength to hard tooth tissues and good adhesion to barium glass filler in the compositions. These problems can be solved by materials based on OCPs.

**Figure 33:**
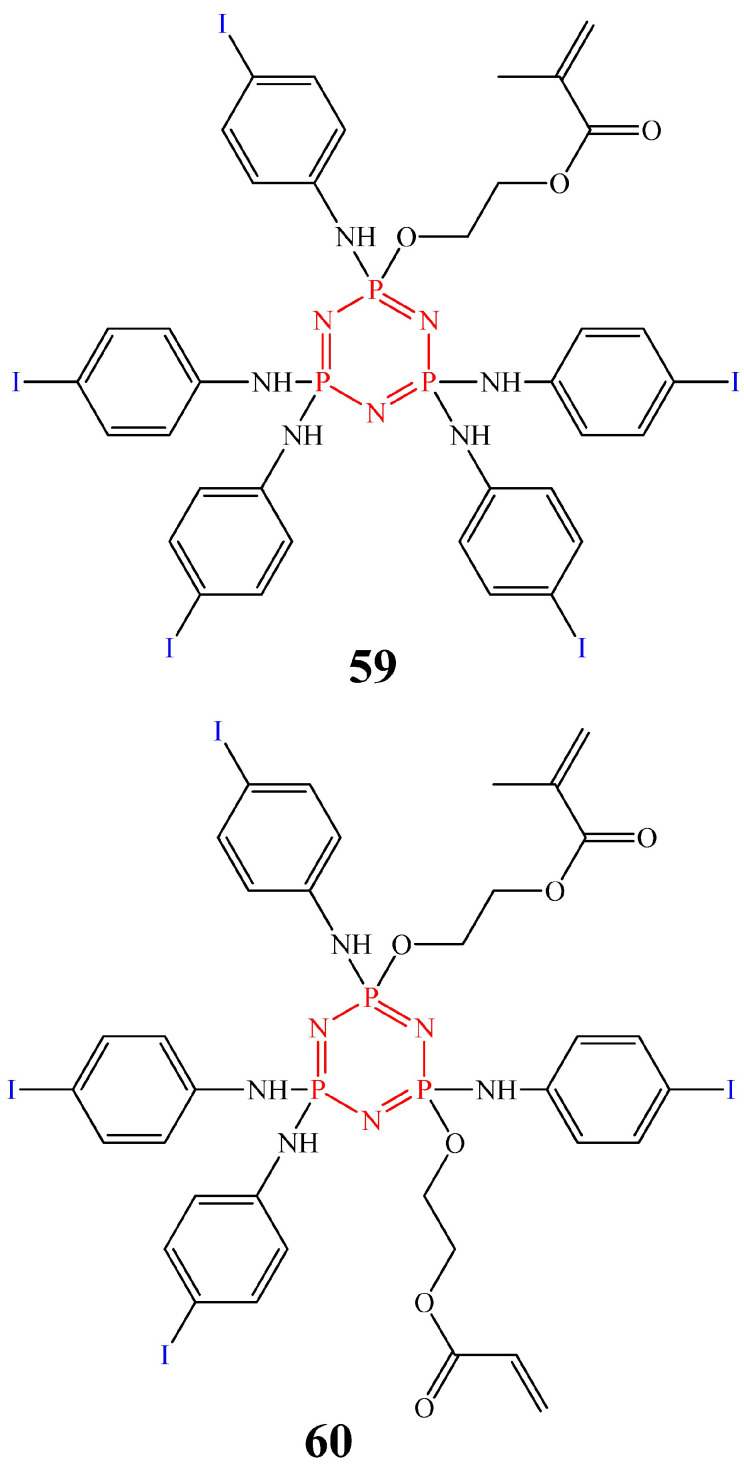
Structure of cyclophosphazene with methacrylate fragments and 4-iodoaniline radicals.

To be used as modifiers of filling materials, OCPs should dissolve well in dental polymer binders, maintain the physical and mechanical properties and esthetics of the composite, and in some cases impart radiopacity, which will allow the compositions to be detected on an X-ray image together with enamel and dentin. For example, cyclophosphazenes **59** and **60** (Figure 33), containing methacrylate fragments and 4-iodoaniline radicals, meet these requirements [87].

The advantage of these iodine-containing OCPs compared to metal oxides and salts (BaO, BaSO4, TiO_2_, SrO, and ZrO_2_) and metal acrylates is their non-toxicity and stable radiopacity, not accompanied by the loss of the radiopaque atom due to hydrolysis, and the absence of a negative effect on the physical and mechanical properties of the composition (flexural strength and elastic modulus) [88]. However, the adhesive properties of the resulting composition have not been studied.

**Figure 34:**
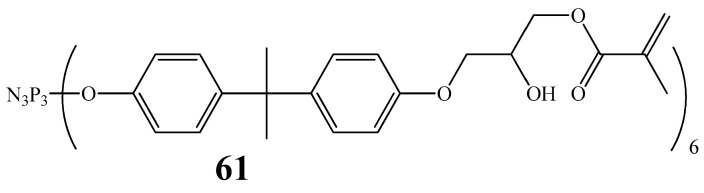
Structure of cyclophosphazene with methacrylic fragments.

To improve the adhesive properties of restorative dental materials, methacrylate-containing phosphazene oligomers (MPOs) consisting of bis-GMA and phosphazene **61** (Figure 34) were developed. These MPOs increased the adhesive strength of cured filled dental compositions to tooth tissues [89,90]. The authors of work [90] explain the increase in the adhesive strength of filled dental compositions as attributable to the chelation of calcium ions Ca^2+^ of hydroxyapatites of enamel and dentin, but do not explain which OCP atoms participate in chelation. A disadvantage of the obtained oligomers is the low content of the phosphazene fraction (less than 50%), as well as the need for multiple rinsing with a solution of potassium carbonate and water during the synthesis of MPO, which may be an obstacle to their production. In addition, to achieve the required adhesive strength, it is necessary to use a large amount of MPOs (10–15 wt.% of the mass of the dental binder bis-GMA/TGM-3).

**Figure 35:**
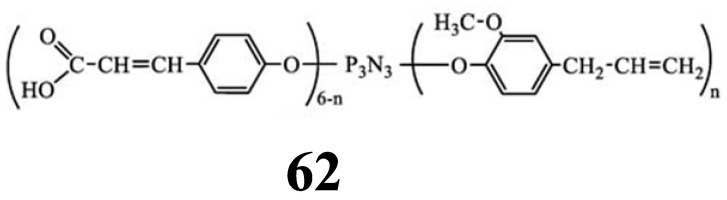
Structure of cyclophosphazene with β-carboxyethenylphenoxy and 4-allyl-2-methoxyphenoxy groups.

Unlike methacrylate-containing cyclophosphazenes, OCPs containing β-carboxyethenyl fragments (compound **62,** Figure 35), due to the presence of carboxyl groups in their composition, react with the hydroxyl groups of hydroxyapatites to form oxygen–calcium ionic bonds, which makes them promising modifiers–adhesives of dental compositions [91]. To improve the solubility of OCPs in the dental methacrylate binder, 4-allyl-2-methoxyphenoxy groups were introduced into its composition. In addition, unlike MPOs, cyclophosphazene **62** is a mixture of homologues with n = 2–4, and not a mixture of phosphazenes with bis-GMA with a low content of phosphazenes in the mixture. As a result, upon adding 10 wt. % of modifier **62** to the filled dental composition based on bis-GMA and TGM-3, an increase in the adhesive strength to dental tissue of more than six times (from 2.5 MPa to 15.4 MPa) was observed.

In addition to the adhesive strength of the composition, the authors of work [91] investigated the effect of the phosphazene modifier on the flexural strength, water absorption, water solubility, and depth of curing, and found that the modified dental composition meets the requirements of ISO 4049:2019 [86] or restorative dental materials. Also, when adding the phosphazene modifier to the composition, an increase in the destructive stress under compression, elastic modulus, and microhardness was observed.

Thus, OCPs containing multiple bonds are promising modifiers of dental restorative materials, since they are embedded in the methacrylate matrix of the polymer binder during radical copolymerization and are not washed out of it into the environment surrounding the dental material, while maintaining the required physical–mechanical (compressive strength, flexural strength, and microhardness) and physical–chemical characteristics (water absorption and water solubility).

Unfortunately, the presented works do not describe the effect of modifiers on the color of restorations, which is very important, since if the material is heavily colored, it will be unsuitable for use as filling compounds.

## 7. Conclusions and Future Perspectives

Despite the disadvantages of OCP-based materials listed in this study, after additional studies, they have been shown to have broad prospects for practical implementation. OCPs containing chalcone groups can be used to treat prostate cancer and ovarian cancer; cyclophosphazenes with pyrrolidine rings—breast cancer, prostate cancer, and colon cancer; cyclophosphazenes with paraben fragments—breast cancer and colon cancer; cyclophosphazenes with anthraquinone fragments—non-small cell lung cancer; cyclophosphazenes with peptide bonds—colon cancer and ovarian cancer; cyclophosphazenes with ferrocene fragments—colon cancer, tuberculosis, and candidiasis; cyclophosphazenes with fluorine atoms—urogenital infections; and phosphazenium salts—liver cancer, lung cancer, and breast cancer. This is due to their monodispersity in contrast to polyphosphazenes. However, most OCPs are poorly soluble in water, which requires the creation of proton salts on their basis, while the mechanism of the antitumor and antimicrobial action of cyclophosphazenes and their salts has not been properly studied. There are also no data on their pharmacokinetics and pharmacodynamics, the effect of cyclophosphazenes on gene expression, or the number and activity of enzymes in tumor tissues, which limits their use in clinical practice.

In addition, OCPs are prone to rapid biodegradation in a physiological environment, which limits their use as PASs. To protect cyclophosphazenes from rapid biodegradation and slow release in physiologically active environments and prolong the circulation of phosphazene in the bloodstream during intravenous administration of the drug, they must be included in nanosized aggregates based on amphiphilic polymers [92]. The formation of hydrogen bonds between the functional groups of cyclophosphazenes with biological activity and the terminal functional groups of the polymer can contribute to slowing the release of cyclophosphazenes from the nanoaggregate and increasing the circulation time of the drug in the vascular bed after injection.

In addition, OCPs are small molecules, which makes it difficult to protect them from degradation by CYP450, other enzymes, or efflux by cell membrane pumps. Also, it is hard to protect their endosome (after they are taken up the cell by endocytosis) from merging with lysosomes. Endosomal escape can be achieved using pH-sensitive materials and nuclear localization signal conjugation [93]. Therefore, further studies of OCP molecules that are capable of nuclear delivery are necessary.

OCPs containing formyl groups are promising compounds for obtaining hydrogels of natural polymers. However, glutaraldehyde is still used as a cross-linking agent, due to its low cost compared to OCPs.

Cyclomatrix phosphazenes and OCPs with donor atoms can be used to manufacture sensors of heavy metals and neurotransmitters in human blood. However, for the use of cyclophosphazenes and cyclomatrix phosphazenes based on them in the field of detection, studies are needed on biochemical samples taken from humans (blood, urine, and saliva). OCPs containing methacrylate, β-carboxyethenylphenoxy, 4-allyl-2-methoxyphenoxy groups, and 4-iodoaniline groups are promising modifiers of polymer dental materials, but this requires studying the color of the materials. This is due to the fact that the color of the material must match the natural color of the teeth and have long-term color stability under the influence of various external factors (dyes in drinks, smoking, and poor oral hygiene, etc.).

Thus, cyclophosphazenes are promising compounds for use in medicine. However, to begin their use in clinical studies in the future, preclinical studies involving laboratory animals are necessary, namely a study of their pharmacodynamic and pharmacokinetic properties and a study of their general toxic properties, local tolerance, toxicokinetics, reproductive toxicity, genotoxicity, and carcinogenicity.

## Figures and Tables

**Table 1:** Molecular docking binding scores of **1**–**5** compounds.

Compound	Hydrogen Bond	π–π Stacking	π–Cation
**1**	ASN18A SER178A TYR224A THR225A	TYR224A	ARG229A
**2**	THR82A ARG229A GLY246B	-	-
**3**	ARG214A GLN336B	HIS393A	LYS352B
**4**	ARG229A	-	ARG229A
**5**	-	TYR108A TRP407A	ARG164B

**Table 2:** MIC and MFC of compound **43** and antibiotics (μmol/L).

Test Microorganisms	Compound 43	Chloramphenicol	Ketoconazole
*B. cereus* (NRRL B-3711)	19.5	1250	-
*B. subtilis* (ATCC 6633)	19.5	78	-
*C. albicans* (ATCC 10231)	19.5	-	1250
*C. krusei* (ATCC 6258)	19.5	-	153
*C. tropicalis* (Y-12968)	19.5	-	1250

## Data Availability

The data presented in this study are available on request from the corresponding author.

## Supplementary Materials

The following supporting information can be downloaded at: https://www.mdpi.com/article/10.3390/biom15020262/s1. Table S1: Minimum concentration values for different cyclophosphazenes and cancer cells; Table S2: MIC, MBC and MFC values of cyclophosphazenes for various microorganisms.

## Abbreviations

The following abbreviations are used in this manuscript:

A2780	Human Ovarian Cancer Cell Line
A549	Human alveolar basal epithelial cell adenocarcinoma
Bis-GMA	2,2-Bis-[p-(3-methacryloyloxy-2-hydroxypropoxy)-phenyl]propane
Bcl-2	Apoptosis regulator, intracellular protein factor
BBB	Blood–brain barrier
BODIPY	4,4-Difluoro-4-bora-3a,4a-diaza-s-indacene
BRAF	Human gene that encodes a protein called B-Raf
Caco-2	Immortalized cell line of human colorectal adenocarcinoma cells
Caspase-3	Proteolytic enzyme
DASD	1,4-Dioxa-8-azaspiro [4,5]decane
DLD-1	Colorectal adenocarcinoma cell line
DNA	Deoxyribonucleic acid
IC_50_	Concentration at which more than 50% of cells are inhibited
FL	Normal human amnion cells
FPP	Hexakis(4-formylphenoxy)cyclotriphosphazene
H1299	Human non-small cell lung carcinoma cell line
HCP	Hexachlorocyclotriphosphazene
HEP-3B	Human hepatocellular carcinoma cell line
HT-29	Human colon cancer cell line
L929	Mouse fibroblast cell line
LNCaP	Lymph node carcinoma of the prostate cell line
MBC	Minimum bactericidal concentration
MCF-7, 4T1	Breast cancer cell lines
MDA-MB-231	Metastatic human breast cancer cell line
MeT-5A	Epithelial cell line from the mesothelium
MIC	Minimum inhibitory concentration
MFC	Minimum fungicidal concentration
MPO	Methacrylate containing phosphazene oligomers
MTT	Colorimetric test for assessing the metabolic activity of cells
OCP	Organocyclophosphazene
PAS	Physiologically active substance
PC-3	Human prostate cancer cell line
RNR	Protein that catalyzes the last step and promotes dNTPs synthesis
ROS	Reactive oxygen species
p53	Cell cycle regulating transcription factor
Src	Protein triosine kinase
TGM-3	Triethyleneglycol dimethacrylate
TS	Thymidylate synthase protein

## References

[1] Ansari L., Shiehzadeh F., Taherzadeh Z., Nikoofal-Sahlabadi S., Momtazi-Borojeni A.A., Sahebkar A., Eslami S. (2017). The most prevalent side effects of pegylated liposomal doxorubicin monotherapy in women with metastatic breast cancer: A systematic review of clinical trials. Cancer Gene Ther..

[2] Chen Y., Wan Y., Wang Y., Zhang H., Jiao Z. (2011). Anticancer efficacy enhancement and attenuation of side effects of doxorubicin with titanium dioxide nanoparticles. Inter. J. Nanomed..

[3] Postma T.J., Vermorken J.B., Liefting A.J.M., Pinedo H.M., Heimans J.J. (1995). Paclitaxel-induced neuropathy. Ann. Oncol..

[4] Lipp H.P., Bokemeyer C., Hartmann J.T., Stanley A. (2003). Cytostatic drugs. Side Eff. Drugs Annu..

[5] Preobrazhenskaya M.N., Shchekotikhin A.E., Shtil A.A., Huang H. (2006). Antitumor anthraquinone analogues for multidrug resistant tumor cells. J. Med. Sci.-Tai..

[6] Ciofu O., Rojo-Molinero E., Macià M.D., Oliver A. (2017). Antibiotic treatment of biofilm infections. Apmis.

[7] Wegrzynowska-Drzymalska K., Mlynarczyk D.T., Chelminiak-Dudkiewicz D., Kaczmarek H., Goslinski T., Ziegler-Borowska M. (2022). Chitosan-Gelatin Films Cross-Linked with Dialdehyde Cellulose Nanocrystals as Potential Materials for Wound Dressings. Int. J. Mol. Sci..

[8] Le Ouay B., Stellacci F. (2015). Antibacterial activity of silver nanoparticles: A surface science insight. Nano Today.

[9] Aguiar F.H., Braceiro A.T., Ambrosano G.M., Lovadino J.R. (2005). Hardness and diametral tensile strength of a hybrid composite resin polymerized with different modes and immersed in ethanol or distilled water media. Dent. Mater..

[10] Tüzüner T., Dimkov A., Nicholson J.W. (2019). The effect of antimicrobial additives on the properties of dental glass-ionomer cements: A review. Acta Biomater. Odontol. Scand..

[11] Promphet N., Ummartyotin S., Ngeontae W., Puthongkham P., Rodthongkum N. (2021). Non-invasive wearable chemical sensors in real-life applications. Anal. Chim. Acta.

[12] Şenkuytu E., Yılmaz S., Çiftçi G.Y. (2023). The new coumarin derivative cyclotetraphosphazene fluorometric chemosensor with reversible, high selective and sensitive for Fe^3+^ ion detection. Inorg. Chim. Acta.

[13] Piskun Y.A., Ksendzov E.A., Resko A.V., Soldatov M.A., Timashev P., Liu H., Vasilenko I.V., Kostjuk S.V. (2023). Phosphazene Functionalized Silsesquioxane-Based Porous Polymer as Thermally Stable and Reusable Catalyst for Bulk Ring-Opening Polymerization of ε-Caprolactone. Polymers.

[14] Bornosuz N.V., Korotkov R.F., Kolenchenko A.A., Shapagin A.V., Orlov A.V., Gorbunova I.Y., Kireev V.V., Sirotin I.S. (2021). The Influence of Substituents in Phosphazene Catalyst-Flame Retardant on the Thermochemistry of Benzoxazine Curing. Polymers.

[15] Das J., Sharma R., Balhara S., Mohanty P. (2023). Thermochemical and electrochemical CO2 reduction by a cyclophosphazene and triazine based inorganic–organic hybrid nanoporous metal-free catalyst. Fuel.

[16] Das J., Rawat S., Maiti A., Singh L., Pradhan D., Mohanty P. (2023). Adsorption of Hg^2+^ on cyclophosphazene and triazine moieties based inorganic-organic hybrid nanoporous materials synthesized by microwave assisted method. Sep. Purif. Technol..

[17] Palabıyık D., Mutlu Balcı C., Beşli S. (2021). The first example of macrocyclic derivatives of cyclotetraphosphazene. Phosphorus Sulfur Silicon Relat. Elem..

[18] Liu L., Bai Y., Ouyang L., Huang L., Shuai Q. (2023). Magnetic phosphazene porous organic polymer for efficient and selective recovery of rare earth elements from acidic wastewater. Chem. Eng. J..

[19] Şahin M.E., Biryan F., Çalışkan E., Koran K. (2024). Coumarin–Phosphazenes: Enhanced Photophysical Properties from Hybrid Materials. Inorg. Chem..

[20] Xu B., Wu M., Liu Y., Wei S. (2023). Study on Flame Retardancy Behavior of Epoxy Resin with Phosphaphenanthrene Triazine Compound and Organic Zinc Complexes Based on Phosphonitrile. Molecules.

[21] Erkovan A.O., Seifi A., Aksoy B.T., Zorlu Y., Khataee A., Çoşut B. (2023). Catalytic Activity of Zn(II) Coordination Polymer Based on a Cyclotriphosphazene-Functionalized Ligand for Removal of Organic Dyes. Catalysts.

[22] Fan W., Li Z., Liao Q., Zhang L., Kong L., Yang Z., Xiang M. (2023). Improving the Heat Resistance and Flame Retardancy of Epoxy Resin Composites by Novel Multifunctional Cyclophosphazene Derivatives. Polymers.

[23] Sarychev I.A., Sirotin I.S., Borisov R.S., Mu J., Sokolskaya I.B., Bilichenko J.V., Filatov S.N., Kireev V.V. (2019). Synthesis of Resorcinol-Based Phosphazene-Containing Epoxy Oligomers. Polymers.

[24] Sirotin I.S., Sarychev I.A., Vorobyeva V.V., Kuzmich A.A., Bornosuz N.V., Onuchin D.V., Gorbunova I.Y., Kireev V.V. (2020). Synthesis of Phosphazene-Containing, Bisphenol A-Based Benzoxazines and Properties of Corresponding Polybenzoxazines. Polymers.

[25] Leng B., Yang J., Zhu C., Wang Z., Shi C., Liu Y., Zhang H., Xu W., Liu B. (2021). Synthesis of a Cyclophosphazene Derivative Containing Multiple Cyano Groups for Electron-Beam Irradiated Flame-Retardant Materials. Polymers.

[26] Mayer-Gall T., Plohl D., Derksen L., Lauer D., Neldner P., Ali W., Fuchs S., Gutmann J.S., Opwis K. (2019). A Green Water-Soluble Cyclophosphazene as a Flame Retardant Finish for Textiles. Molecules.

[27] Stakhanov A.I., Elmanovich I.V., Kravchenko E.I., Khakina E.A., Pavlov A.A., Kharitonova E.P., Gallyamov M.O. (2023). New fluorinated cyclophosphazenes: Synthesis, properties, applications. Phosphorus Sulfur Silicon Relat. Elem..

[28] Pilch-Pitera B., Czachor-Jadacka D., Byczyński Ł., Dutkiewicz M., Januszewski R., Kowalczyk K., Nowak W.J., Pojnar K. (2024). Hexakis[p-(hydroxymethyl)phenoxy]cyclotriphosphazene as an Environmentally Friendly Modifier for Polyurethane Powder Coatings with Increased Thermal Stability and Corrosion Resistance. Materials.

[29] Blackstone V., Lough A.J., Murray M., Manners I. (2009). Probing the Mechanism of the PCl_5_− Initiated Living Cationic Polymerization of the Phosphoranimine Cl_3_P=NSiMe_3_ using Model Compound Chemistry. J. Am. Chem. Soc..

[30] Gholamrezazadeh C., Hakimi M., Dadmehr M. (2024). Synthesis, characterization and biological activities of nitrophenol-substituted phosphazene dendrimers. J. Mol. Struct..

[31] Wang L., Yang Y.X., Shi X., Mignani S., Caminade A.M., Majoral J.P. (2018). Cyclotriphosphazene core-based dendrimers for biomedical applications: An update on recent advances. J. Mater. Chem. B.

[32] Gascón E., Maisanaba S., Otal I., Valero E., Repetto G., Jones P.G., Jiménez J. (2020). (Amino) cyclophosphazenes as multisite ligands for the synthesis of antitumoral and antibacterial silver (I) complexes. Inorg. Chem..

[33] Casella G., Carlotto S., Lanero F., Mozzon M., Sgarbossa P., Bertani R. (2022). Cyclo- and Polyphosphazenes for Biomedical Applications. Molecules.

[34] Chen F., Teniola O.R., Ogueri K.S. (2023). Recent Trends in the Development of Polyphosphazenes for Bio-applications. Regen. Eng. Transl. Med..

[35] Jin G.-W., Rejinold N.S., Choy J.-H. (2022). Polyphosphazene-Based Biomaterials for Biomedical Applications. Int. J. Mol. Sci..

[36] Dagdag O., Kim H. (2024). Progress in the Field of Cyclophosphazenes: Preparation, Properties, and Applications. Polymers.

[37] Bhavsar D.B., Patel V., Sawant K.K. (2020). Design and characterization of dual responsive mesoporous silica nanoparticles for breast cancer targeted therapy. Eur. J. Pharm. Sci..

[38] Doğan H., Bahar M.R., Çalışkan E., Tekin S., Uslu H., Akman F., Koran K., Sandai S., Görgülü A.O. (2022). Synthesis and spectroscopic characterizations of hexakis [(1-(4′-oxyphenyl)-3-(substituted-phenyl) prop-2-en-1-one)] cyclotriphosphazenes: Their in vitro cytotoxic activity, theoretical analysis and molecular docking studies. J. Biomol. Struct. Dynam..

[39] Beytur A., Tekin Ç., Çalışkan E., Tekin S., Koran K., Görgülü A.O., Sandal S. (2022). Hexa-substituted cyclotriphosphazene derivatives containing hetero-ring chalcones: Synthesis, in vitro cytotoxic activity and their DNA damage determination. Bioorg. Chem..

[40] Labarre J.F., Faucher J.P., Levy G., Sournies F., Cros S., François G. (1979). Antitumour activity of some cyclophosphazenes. Eur. J. Cancer.

[41] Brandt K., Kruszynski R., Bartczak T.J., Porwolik-Czomperlik I. (2001). AIDS-related lymphoma screen results and molecular structure determination of a new crown ether bearing aziridinylcyclophosphazene, potentially capable of ion-regulated DNA cleavage action. Inorg. Chim. Acta.

[42] Hakimi M., Rezaei H., Moeini K., Mardani Z., Carpenter-Warren C. (2020). Solvent free synthesis of three cyclotriphosphazene derivatives containing piperazine substituents using microwave irradiation. Spectral, theoretical, solution and docking studies. Phosphorus Sulfur Silicon Relat. Elem.

[43] Okumuş A., Akbaş H., Kılıç Z., Yasemin Koç L., Açık L., Aydın B., Dal H. (2016). Phosphorus–nitrogen compounds part 33: In vitro cytotoxic and antimicrobial activities, DNA interactions, syntheses, and structural investigations of new mono (4-nitrobenzyl) spirocyclotriphosphazenes. Res. Chem. Inter..

[44] Akbaş H., Şenocak A., Kılıç Z., Tayhan S.E., Bilgin S., Yıldırım A., Hökelek T. (2023). Syntheses of tetrachloro and tetraamino (2-furanylmethyl) spiro (N/N) cyclotriphosphazenes: Chemical, structural elucidation, antiproliferative and antimigratory activity studies. J. Mol. Struct..

[45] Akbaş H., Şenocak A., Kılıç Z., Erden Tayhan S., Bilgin S., Yıldırım Kocaman A., Hökelek T. (2023). Bis (2-furanylmethyl) monospiro (N/N) cyclotriphosphazenes: Synthesis, structural characterization, antiproliferative, and antimigratory activity studies. Phosphorus Sulfur Silicon Relat. Elem..

[46] Şenkuytu E., Akbaş N., Yıldırım T., Çiftçi G.Y. (2022). The bioactive new type paraben decorated dispiro-cyclotriphosphaze compounds: Synthesis, characterization and cytotoxic activity studies. J. Mol. Struct..

[47] Çiftçi G.Y., Bayık N., Turhal G., Baslilar İ.N., Demiroglu-Zergeroglu A. (2022). The first mono anthraquinone substituted monospiro cyclotriphosphazene derivatives and their effects on non-small cell lung cancer cells. Inorg. Chim. Acta.

[48] Thorn C.F., Oshiro C., Marsh S., Hernandez-Boussard T., McLeod H., Klein T.E., Altman R.B. (2011). Doxorubicin pathways: Pharmacodynamics and adverse effects. Pharmacog. Genom..

[49] Koran K., Çalışkan E., Öztürk D.A., Çapan İ., Tekin S., Sandal S., Görgülü A.O. (2023). The first peptide derivatives of dioxybiphenyl-bridged spiro cyclotriphosphazenes: In vitro cytotoxicity activities and DNA damage studies. Bioorg. Chem..

[50] Chinnadurai R.K., Khan N., Meghwanshi G.K., Ponne S., Althobiti M., Kumar R. (2023). Current research status of anti-cancer peptides: Mechanism of action, production, and clinical applications. Biomed. Pharmacother..

[51] Xie M., Liu D., Yang Y. (2020). Anti-cancer peptides: Classification, mechanism of action, reconstruction and modification. Open Biol..

[52] Okumuş A., Elmas G., Kılıç Z., Binici A., Ramazanoğlu N., Açık L., Şimşek H. (2021). The comparative reactions of 2-cis-4-ansa and spiro cyclotetraphosphazenes with difunctional ligands: Structural and stereogenic properties, electrochemical, antimicrobial and cytotoxic activity studies. Appl. Organometal. Chem..

[53] Elmas G., Okumuş A., Cemaloğlu R., Kılıç Z., Çelik S.P., Açık L., Hökelek T. (2017). Phosphorus-nitrogen compounds. part 38. Syntheses, characterizations, cytotoxic, antituberculosis and antimicrobial activities and DNA interactions of spirocyclotetraphosphazenes with bis-ferrocenyl pendant arms. J. Organometal. Chem..

[54] Ornelas C., Astruc D. (2023). Ferrocene-Based Drugs, Delivery Nanomaterials and Fenton Mechanism: State of the Art, Recent Developments and Prospects. Pharmaceutics.

[55] Okumuş A., Akbaş H., Karadağ A., Aydın A., Kılıç Z., Hökelek T. (2017). Antiproliferative Effects against A549, Hep3B and FL Cell Lines of Cyclotriphosphazene-Based Novel Protic Molten Salts: Spectroscopic, Crystallographic and Thermal Results. ChemistrySelect.

[56] Elmas G., Okumuş A., Kilic Z., Özbeden P., Acik L., Çağdaş Tunalı B., Hökelek T. (2020). Phosphorus-nitrogen compounds. Part 48. Syntheses of the phosphazenium salts containing 2-pyridyl pendant arm: Structural characterizations, thermal analysis, antimicrobial and cytotoxic activity studies. Indian J. Chem. A.

[57] Ozsoy F., Ozay O. (2023). Enhanced drug carrier capacity after post modification process for amino acid substitute phosphazene based microspheres with anticancer activity. J. Drug. Deliv. Sci. Technol..

[58] Gaumet M., Vargas A., Gurny R., Delie F. (2008). Nanoparticles for drug delivery: The need for precision in reporting particle size parameters. Eur. J. Pharm. Biopharm..

[59] Ozsoy F., Onder F.C., Ilgin P., Ozay H., Onder A., Ozay O. (2023). Self-assembled silibinin-containing phosphazene/cystamine hybrid nanospheres as biodagradable dual-drug carriers with improved anticancer activity on a breast cancer cell line. Mater. Today Commun..

[60] Jeyamogan S., Khan N.A., Siddiqui R. (2021). Application and Importance of Theranostics in the Diagnosis and Treatment of Cancer. Arch. Med. Res..

[61] Kwon N., Kim K.H., Park S., Cho Y., Park E.Y., Lim J., Yoon J. (2022). Hexa-BODIPY-cyclotriphosphazene based nanoparticle for NIR fluorescence/photoacoustic dual-modal imaging and photothermal cancer therapy. Biosen. Bioelectron..

[62] Bludau H., Czapar A.E., Pitek A.S., Shukla S., Jordan R., Steinmetz N.F. (2017). POxylation as an alternative stealth coating for biomedical applications. EPJ.

[63] Stephanie F., Saragih M., Tambunan U.S.F. (2021). Recent Progress and Challenges for Drug-Resistant Tuberculosis Treatment. Pharmaceutics.

[64] Ardhianto D., Suharjono, Soedarsono, Fatmawati U. (2021). Analysis of the side effect of QTc interval prolongation in the bedaquiline regimen in drug resistant tuberculosis patients. J. Basic Clin. Physiol. Pharmacol..

[65] Binici A. (2022). Syntheses of Hexaminomonoferrocenylspiro (N/O) cyclotetraphosphazenes: Spectral Properties and Antituberculosis Activities. CSJ.

[66] Binici A., Okumuş A., Elmas G., Kılıç Z., Ramazanoğlu N., Açık L., Hökelek T. (2019). Phosphorus–nitrogen compounds. Part 42. The comparative syntheses of 2-cis-4-ansa (N/O) and spiro (N/O) cyclotetraphosphazene derivatives: Spectroscopic and crystallographic characterization, antituberculosis and cytotoxic activity studies. New J. Chem..

[67] Kumari A., Singh R.K. (2020). Morpholine as ubiquitous pharmacophore in medicinal chemistry: Deep insight into the structure-activity relationship (SAR). Bioorg. Chem..

[68] Okumuş A., Elmas G., Cemaloğlu R., Aydın B., Binici A., Şimşek H., Hökelek T. (2016). Phosphorus–nitrogen compounds. Part 35. Syntheses, spectroscopic and electrochemical properties, and antituberculosis, antimicrobial and cytotoxic activities of mono-ferrocenyl-spirocyclotetraphosphazenes. New J. Chem..

[69] Zhang T., Guo J., Ding Y., Mao H., Yan F. (2019). Redox-responsive ferrocene-containing poly (ionic liquid) s for antibacterial applications. Sci. China Chem..

[70] Elmas G., Kılıç Z., Çoşut B., Keşan G., Açık L., Cam M., Hökelek T. (2021). Synthesis of Bis (2,2,3,3-tetrafluoro-1,4-butanedialkoxy)-2-trans-6-bis (4-fluorobenzyl) spirocyclotetraphosphazene: Structural Characterization, Biological Activity and DFT Studies. J. Chem. Crystallogr..

[71] Okumuş A., Elmas G., Binici A., Aydın B., Açık L., Kılıç Z., Hökelek T. (2022). Phosphorus-nitrogen compounds. Part 62. Preparation of tetraaminobis (4-fluorobenzyl) spiro (N/N) cyclotriphosphazenes: Chemical, structural characterizations, antimicrobial, antioxidant and DNA-binding activity studies. Inorg. Chim. Acta..

[72] Kaygusuz Ö., Çetin G., Türkyılmaz O., Darcan C., Ture S. (2020). The reactions of hexachlorocyclotriphosphazene with piperidine, N-(1-naphthyl) ethylenediamine and 2-(2-hydroxyethylamino) ethanol. Antimicrobial activities of iminodiethoxy-substituted cyclotriphosphazene derivatives. Phosphorus Sulfur Silicon Relat. Elem..

[73] Ture S., Darcan C., Türkyılmaz O., Kaygusuz Ö. (2020). Synthesis, structural characterization and antimicrobial activities of cyclochlorotriphosphazene derivatives derived from N-(1-Naphthyl) ethylenediamine. Phosphorus Sulfur Silicon Relat. Elem..

[74] Wang B., Pachaiyappan B., Gruber J.D., Schmidt M.G., Zhang Y.M., Woster P.M. (2016). Antibacterial diamines targeting bacterial membranes. J. Med. Chem..

[75] Ozay H., Ilgin P., Ozay O. (2021). Novel hydrogels based on crosslinked chitosan with formyl-phosphazene using Schiff-base reaction. Int. J. Polym. Mater..

[76] Gholivand K., Mohammadpour M., Derakhshankhah H., Samadian H., Aghaz F., Malekshah R.E., Rahmatabadi S. (2023). Composites based on alginate containing formylphosphazene-crosslinked chitosan and its Cu (II) complex as an antibiotic-free antibacterial hydrogel dressing with enhanced cytocompatibility. Int. J. Biol. Macromol..

[77] Yudaev P., Butorova I., Chuev V., Posokhova V., Klyukin B., Chistyakov E. (2023). Wound gel with antimicrobial effects based on polyvinyl alcohol and functional aryloxycyclotriphosphazene. Polymers.

[78] Qiu J., Wang Y., Liu Y., Zhang M., Wu Z., Liu C. (2015). A Ph-sensitive drug carrier based on maleic acid-substituted cyclotriphosphazene. Phosphorus Sulfur Silicon Relat. Elem..

[79] Uslu A., Tümay S.O., Yeşilot S. (2022). Fluorescent materials based on phosphazene derivatives and their applications: Sensors and optoelectronic devices. J. Photochem. Photobio C.

[80] Örüm S.M., Demircioğlu Y.S. (2018). Crosslinked polyphosphazene nanospheres with anticancer quercetin: Synthesis, spectroscopic, thermal properties, and controlled drug release. Macromol. Res..

[81] Abbas Y., Akhtar N., Ghaffar S., Al-Sulami A.I., Asad M., Mazhar M.E., Wu Z. (2022). Cyclophosphazene Intrinsically Derived Heteroatom (S,N,P,O)-Doped Carbon Nanoplates for Ultrasensitive Monitoring of Dopamine from Chicken Samples. Biosensors.

[82] Yang Z., She M., Yin B., Cui J., Zhang Y., Sun W., Li J., Shi Z. (2012). Three Rhodamine-based “off–on” Chemosensors with high selectivity and sensitivity for Fe3+ imaging in living cells. J. Org. Chem..

[83] Kagit R., Yildirim M., Ozay O., Yesilot S., Ozay H. (2014). Phosphazene based multicentered naked-eye fluorescent sensor with high selectivity for Fe^3+^ ions. Inorg. Chem..

[84] Yin W., Cui H., Yang Z., Li C., She M., Yin B., Li J., Zhao G., Shi Z. (2011). Facile synthesis and characterization of rhodamine-based colorimetric and “off–on” fluorescent chemosensor for Fe^3+^. Sens. Actuators B Chem..

[85] Chistyakov E., Yudaev P., Nelyubina Y. (2022). Crystallization of Nano-Sized Macromolecules by the Example of Hexakis-[4-{(N-Allylimino)methyl}phenoxy]cyclotriphosphazene. Nanomaterials.

[86] (2019). Dentistry–Polymer-Based Restorative Materials.

[87] Zhao Y., Lan J., Wang X., Deng X., Cai Q., Yang X. (2014). Synthesis of iodine-containing cyclophosphazenes for using as radiopacifiers in dental composite resin. Mater. Sci. Eng. C.

[88] He J., Söderling E., Lassila L.V., Vallittu P.K. (2012). Incorporation of an antibacterial and radiopaque monomer in to dental resin system. Dent. Mater..

[89] Sirotin I.S., Son V.X., Bilichenko Y.V., Borisov R.S., Gorbunova E.A., Kireev V.V. (2022). Methacrylate-Containing Phosphazene Oligomers. Polym. Sci. Ser. B.

[90] Bilichenko Y.V., Son V., Thuan P., Sirotin I.S., Kireev V.V., Chuev V.P., Klyukin B.V., Posohova V.F. (2022). Synthesis of phosphazene methacrylate oligomers and their use for modification of dental composite materials. Plast. Massy..

[91] Chistyakov E.M., Kolpinskaya N., Posokhova V., Chuev V. (2020). Dental Composition Modified with Aryloxyphosphazene Containing Carboxyl Groups. Polymers.

[92] Luss A.L., Kulikov P.P., Romme S.B., Andersen C.L., Pennisi C.P., Docea A.O., Kuskov A.N., Velonia K., Mezhuev Y.O., Shtilman M.I. (2018). Nanosized carriers based on amphiphilic poly-N-vinyl-2-pyrrolidone for intranuclear drug delivery. Nanomedicine.

[93] Roy S.M., Garg V., Sivaraman S.P., Barman S., Ghosh C., Bag P., Mohanasundaram P., Maji P.S., Basu A., Dirisala A. (2023). Overcoming the barriers of nuclear-targeted drug delivery using nanomedicine-based strategies for enhanced anticancer therapy. J. Drug Deliv. Sci. Technol..

