# Supplementary material for: Organocyclophosphazenes and Materials Based on Them for Pharmaceuticals and Biomedicine
Source: Biomolecules. 2025 Feb 11;15(2):262. doi: 10.3390/biom15020262 (PMC11852944; doi:10.3390/biom15020262)
Supplement: Supplementary file 1 [file biomolecules-15-00262-s001.zip › biomolecules-3443542-supplementary.pdf]

Table S1. Minimum concentration values for different cyclophosphazenes and cancer cells.

**Cyclophosphazenes**	**Minimum concentration, μmol/l**	**Cancer cells**	**References**
Compound **1**	25	A2780	[38]
	100	LNCaP	
	1	PC-3	
Compound **2**	100	A2780	
	25	LNCaP	
	1	PC-3	
Compound **3**	100	A2780	
	50	LNCaP	
	50	PC-3	
Compound **4**	50	A2780	
	50	LNCaP	
	1	PC-3	
Compound **5**	1	A2780	
	1	LNCaP	
	5	PC-3	
Compound **6**	25	A2780	[39]
	5	LNCaP	
	1	PC-3	
Compound **7**	25	A2780	
	1	LNCaP	
	1	PC-3	
Compound **8**	1	A2780	
	25	LNCaP	
	1	PC-3	
Compound **9**	1	A2780	
	1	LNCaP	
	1	PC-3	
Compound **10**	50	MDA-MB-231	[43]
Compound **11**	25		
Compound **12**	3.12	MCF-7	[44]

	3.12	HT-29	
	6.25	PC-3	
Compound **13**	3.12	MCF-7	
	3.12	HT-29	
	3.12	PC-3	
Compound **14**	3.12	MCF-7	
	6.25	HT-29	
	25	PC-3	
Compound **15**	3.12	MCF-7	
	3.12	HT-29	
	3.12	PC-3	
Compound **16**	200	MCF-7	[46]
	100	DLD-1	
Compound **17**	12.5	MCF-7	
	200	DLD-1	
Compound **18**	50	MCF-7	
	200	DLD-1	
Compound **19**	50	MCF-7	
	100	DLD-1	
Compound **20**	50	MCF-7	
	100	DLD-1	
Compound **21**	100	A549	[47]
	100	H1299	
Compound **22**	50	A549	
	50	H1299	
Compound **23**	100	A549	
	100	H1299	
Compound **24**	1	A2780	[49]
	1	Caco-2	
Compound **25**	1	A2780	
	1	Caco-2	
Compound **26**	25	A2780	
	25	Caco-2	
Compound **27**	1	A2780	
	1	Caco-2	

Compound **28**	1	A2780	
	100	PC-3	
Compound **29**	1	A2780	
	100	PC-3	
Compound **30**	1	A2780	
	100	PC-3	
Compound **31**	1	A2780	
	100	PC-3	
Compound **32**	200	MCF7	[52]
Compound **33**	200		
Compound **34**	50	DLD-1	[53]
Compound **35**	12.5		
Compound **36**	50		

Table S2. MIC, MBC and MFC values of cyclophosphazenes for various microorganisms.

**Cyclophosphazenes**	**Microorganisms**	**MIC, µg/ml**	**MBC, µg/ml**	**MFC, µg/ml**	**References**
Compound **32**	*Mycobacterium Tuberculosis*	80	-	-	[65]
Compound **35**		30	-	-	[53]
Compound **36**		16	-	-	
Compound **37**		35	-	-	[65]
Compound **38**		70	-	-	
Compound **39**		38	-	-	[66]
Compound **40**		36	-	-	
Compound **41**		3	-	-	[68]
Compound **42**		3	-	-	
Compound **43**		3	-	-	
Compound **43**	*B. cereus, B. subtilis, E. faecalis,*	-	19.5	-	[68]
	C. albicans, C. krusei, C. tropicalis	-	-	19.5	
Compound **44**	*B. subtilis*	25.75	25.75	-	[52]
	P. vulgaris	13	13	-	
	K. pneumaniae	6.5	6.5	-	
Compound **45**	*E. faecalis,*	2000	>2000	-	[70]
	S. aureus	125	125	-	
	K. pneumoniae	500	500	-	
	B. cereus	2000	>2000	-	
Compound **46**	*E. faecalis,*	2500	2500	-	[71]
	S. aureus	2500	>2500	-	
	K. pneumoniae	2500	2500	-	
	B. cereus	625	2500	-	
	B. subtilis	625	>2500	-	
Compound **47**	*E. faecalis,*	625	>2500	-	
	S. aureus	2500	2500	-	

	K. pneumoniae	625	2500	-	
	B. cereus	625	1250	-	
	B. subtilis	625	>2500	-	
Compound **48**	*E. faecalis,*	625	>2500	-	
	S. aureus	1250	>2500	-	
	K. pneumoniae	625	2500	-	
	B. cereus	625	2500	-	
	B. subtilis	625	>2500	-	
Compound **49**	*E. coli*	208	250	-	[72]
	S. aureus	333	416	-	
	C. albicans	166	-	250	
Compound **50**	*E. coli*	1039	1662	-	
	S. aureus	831	2494	-	
	C. albicans	416	-	831	
Compound **51**	*E. coli*	65000	65000	-	
	S. aureus	254	508	-	
	C. albicans	127	-	254	
Compound **52**	*E. coli*	4938	9875	-	
	S. aureus	2963	5925	-	
	C. albicans	2963		2963	
Compound **53**	*E. coli*	4388	8775	-	
	S. aureus	2193	7313	-	
	C. albicans	1828	-	2194	
Compound **54**	*E. coli*	3750	3750	-	
	S. aureus	7500	10000	-	
	C. albicans	2500	-	2500	
Compound **55**	*E. coli*	14	45	-	
	S. aureus	16	36	-	
	C. albicans	9	-	14	
Compound **56**	*E. coli*	8	10	-	[73]
	S. aureus	6	12	-	
	C. albicans	4	-	40	
